# Supplementary material for: Decision making biases in the allied health professions: A systematic scoping review
Source: PLoS One. 2020 Oct 20;15(10):e0240716. doi: 10.1371/journal.pone.0240716 (PMC7575084; doi:10.1371/journal.pone.0240716)
Supplement: S2 File — (DOCX) [file pone.0240716.s002.docx]

**Supporting Information**

**S2 Definition for allied health professionals included in the review.**

The definition ascribed by Allied Health Professions Australia (AHPA), alongside the list of professions can be found at https://ahpa.com.au/what-is-allied-health/

An allied health profession is one which has:

- a direct patient care role and may have application to broader public health outcomes;
- a national professional organisation with a code of ethics/conduct and clearly defined membership requirements;
- university health sciences courses (not medical, dental or nursing) at AFQ Level 7 or higher, accredited by their relevant national accreditation body;
- clearly articulated national entry level competency standards and assessment procedures;
- a defined core scope of practice;
- robust and enforceable regulatory mechanisms.

Furthermore, the profession must consist of allied health professionals who:

- are autonomous practitioners;
- practice in an evidence-based paradigm, using an internationally recognised body of knowledge to protect, restore and maintain optimal physical, sensory, psychological, cognitive, social and cultural function;
- may utilise or supervise assistants, technicians and support workers.

The AHPA includes the following professions under this definition:

- Arts Therapy
- Audiology
- Chiropractic
- Dietetics
- Exercise Physiology
- Genetic Counselling
- Music Therapy
- Orthotics/Prosthetics
- Osteopathy
- Perfusion
- Physiotherapy (Physical Therapy)
- Podiatry
- Psychology
- Speech Pathology
- Rehabilitation Counselling
- Social Work
- Sonography
