# Supplementary material for: Decision making biases in the allied health professions: A systematic scoping review
Source: PLoS One. 2020 Oct 20;15(10):e0240716. doi: 10.1371/journal.pone.0240716 (PMC7575084; doi:10.1371/journal.pone.0240716)
Supplement: S3 File — (DOCX) [file pone.0240716.s003.docx]

**Supporting Information**

**S3 Example database search strategy.**

| # | **Searches** |
| --- | --- |
| 1 | exp Decision Making/ |
| 2 | decision*.ti,ab. |
| 3 | diagnosis.ti,ab. |
| 4 | diagnostic.ti,ab. |
| 5 | clinical assessment.ti,ab. |
| 6 | goal setting.ti,ab. |
| 7 | care planning.ti,ab. |
| 8 | goal planning.ti,ab. |
| 9 | treatment.ti,ab. |
| 10 | or/1-9 |
| 11 | exp Health Personnel/ |
| 12 | social work*.ti,ab. |
| 13 | art therap*.ti,ab. |
| 14 | physiotherap*.ti,ab. |
| 15 | physical therap*.ti,ab. |
| 16 | speech therap*.ti,ab. |
| 17 | chiropract*.ti,ab. |
| 18 | audiolog*.ti,ab. |
| 19 | audiometr*.ti,ab. |
| 20 | hearing care.ti,ab. |
| 21 | medicine.ti,ab. |
| 22 | medical.ti,ab. |
| 23 | clinician*.ti,ab. |
| 24 | optom*.ti,ab. |
| 25 | optic*.ti,ab. |
| 26 | vision.ti,ab. |
| 27 | optha*.ti,ab. |
| 28 | dietician*.ti,ab. |
| 29 | dietetics.ti,ab. |
| 30 | speech patholog*.ti,ab. |
| 31 | eye care.ti,ab. |
| 32 | (counselling or counseling).ti,ab. |
| 33 | music therap*.ti.,b |
| 34 | exercise physiolog*.ti.ab. |
| 35 | occupational therap*.ti.ab. |
| 36 | orthoptics.ti.ab. |
| 37 | orthotics.ti.ab. |
| 38 | prosthetics.ti.ab. |
| 39 | osteopath*.ti.ab. |
| 40 | perfusion.ti.ab. |
| 41 | podiatr*.ti.ab |
| 42 | sonograph*.ti.ab. |
| 43 | or/11-42 |
| 44 | (reasoning adj1 error*).ti,ab. |
| 45 | (cognitive adj1 bias*).ti,ab. |
| 46 | (cognitive adj1 error*).ti,ab. |
| 47 | heuristic*.ti,ab. |
| 48 | dual process.ti,ab. |
| 50 | (logic* adj2 error*).ti,ab. |
| 51 | (anchoring adj1 bias*).ti,ab. |
| 52 | (availability adj1 bias*).ti,ab. |
| 53 | (confirmation adj1 bias*).ti,ab. |
| 54 | (commission adj1 bias*).ti,ab. |
| 55 | (decoy adj1 effect*).ti,ab. |
| 56 | (fundamental adj1 attribution adj1 error*).ti,ab. |
| 57 | (gambl* adj1 bias*).ti,ab. |
| 58 | (hindsight adj1 bias*).ti,ab. |
| 59 | (impact adj1 bias*).ti,ab. |
| 60 | (loss adj1 aversion adj1 bias*).ti,ab. |
| 61 | (affect* adj1 bias*).ti,ab. |
| 62 | (mood adj1 bias*).ti,ab. |
| 63 | (forecasting adj1 bias*).ti,ab. |
| 64 | (normaliz* adj1 bias*).ti,ab. |
| 65 | (ambiguity adj1 effect*).ti,ab. |
| 66 | (bandwagon adj1 effect*).ti,ab. |
| 67 | (conjunction adj1 fallacy).ti,ab. |
| 68 | (context adj1 effect*).ti,ab. |
| 69 | (contrast adj1 effect*).ti,ab. |
| 70 | (curiosity adj1 bias*).ti,ab. |
| 71 | (default adj1 bias*).ti,ab. |
| 72 | (egocentric adj1 bias*).ti,ab. |
| 73 | (endowment adj1 effect*).ti,ab. |
| 74 | (focusing adj1 effect*).ti,ab. |
| 75 | (framing adj2 effect*).ti,ab. |
| 76 | (information adj1 bias*).ti,ab. |
| 77 | (omission adj1 bias*).ti,ab. |
| 78 | (optimism adj1 bias*).ti,ab. |
| 79 | (order adj1 bias*).ti,ab. |
| 80 | (outcome adj1 bias*).ti,ab. |
| 81 | (overconfidence adj1 bias*).ti,ab. |
| 82 | (primacy adj1 effect*).ti,ab. |
| 83 | (recall adj1 bias*).ti,ab. |
| 84 | (recency adj1 effect*).ti,ab. |
| 85 | (representativ* adj1 bias*).ti,ab. |
| 86 | (self enhanc* adj1 bias*).ti,ab. |
| 87 | (status adj1 quo adj1 bias*).ti,ab. |
| 88 | (sunk adj1 cost adj1 bias*).ti,ab. |
| 89 | (stereotyp* adj1 bias*).ti,ab. |
| 90 | (affective adj1 bias*).ti.ab. |
| 91 | (represent* adj1 heuristic*).ti,ab. |
| 92 | (availability adj1 heuristic*).ti,ab. |
| 93 | (anchoring adj1 heuristic*).ti,ab. |
| 94 | (affect adj1 heuristic*).ti,ab. |
| 95 | or/44-94 |
| 96 | 10 and 43 and 95 |

*Note.* This example is adapted for (Ovid MEDLINE(R) Epub Ahead of Print, In-Process & Other Non-Indexed Citations, Ovid MEDLINE(R) Daily and Ovid MEDLINE(R) 1946 to Present). Strategies for other databases available upon request.
