# Supplementary material for: Decision making biases in the allied health professions: A systematic scoping review
Source: PLoS One. 2020 Oct 20;15(10):e0240716. doi: 10.1371/journal.pone.0240716 (PMC7575084; doi:10.1371/journal.pone.0240716)
Supplement: S4 File — (DOC) [file pone.0240716.s004.doc]

**Supporting Information**

**S4 Summary of the included studies (n = 149)**

| **Authors, Year, Country** | **Type of Publication** | **Allied Health Profession** | **Participants (n)** | **Study Design and Brief Study Description** | **Decision Type** | **Predictions for Relevant Outcomes Representing the Bias** | **Type of Bias/es** | **Bias/es detected for at least one outcome**  **(Y/N)** |
| --- | --- | --- | --- | --- | --- | --- | --- | --- |
| Abramowitz, 1976; USA^1^ | Journal Article | Psychology | Group therapists (n = 122) | Participants read one version of a set of case materials that varied only by sex of the depicted client (female/male). Participants were asked to provide clinical reactions to the case materials. Participants were also assessed and grouped by ‘traditional’ or ‘non-traditional’ psychotherapy leaning. | Assessment | Predicted that clinical assessments and responses to the client would differ with the sex assigned to the case character and traditional/non-traditional leaning of the participant. | Sex bias | Y |
| Abreu, 1999; USA^2^ | Journal Article | Psychology | Licensed psychologists; clinical and counselling graduate students (n = 60) | Participants were allocated to a high or low priming condition. Two lists of 100 words were developed, with either 80% African American related words (high priming) or 80% neutral words (low priming). Words were flashed, one at a time for 80 ms, in front of participants. Participants were then asked to read a brief clinical description and therapy session excerpt describing mixed clinical features. Participants were asked to complete a clinical impressions rating scale and answer questions relating to diagnosis. | Diagnosis | Predicted that participants in the high priming condition would respond to the rating task with more negative evaluations on dimensions considered more relevant to African Americans. | Racial bias | Y |
| Aronoff, 1997; Canada^3^ | PhD Dissertation | Psychology | Professional counsellors (n = 40) | Participants were assigned to one of four case file vignettes containing identical information, but presented in a different order. Participants completed a questionnaire relating to the client information and were asked to assess client functioning. Participants also undertook a ‘think aloud’ interview to determine the clinical inference process. | Assessment | Order of information presented in case files predicted to alter assessment of client functioning. | Anchoring; Fundamental attribution error | Y |
| Babab et al., 1975; Israel^4^ | Journal Article | Psychology | Education psychology graduate students (n = 18) | Participants were asked to score a Wechsler Intelligence Scale for Children (WISC) record of an Israeli fifth grader. The same record was given to all participants with one of two cover sheets portraying either an underachieving disadvantaged child or  a high-achieving, upper-middle class child. | Assessment | Predicted that average WISC score would differ depending on the information on the cover sheet: underachieving disadvantaged versus high-achieving, upper-middle class child. | Experimenter bias; Expectancy bias; Socioeconomic status bias | Y |
| Barkin, 1991; USA^5^ | PhD Dissertation | Psychology | Psychologists (n = 90) | Participants were shown one version of a videotape of a hypothetical patient. Versions were modified such that physical health status (HIV positive/terminal cancer/no diagnosed medical condition) and sexual preference (heterosexual/homosexual) were randomised across the versions. Participants then completed a 19-item questionnaire to assess judgements relating to diagnosis, prognosis and personal judgements about ability to treat the patient. | Diagnosis; Prognosis | Predicted that health status and sexual preference of the patient would be reflected in changes in diagnosis and prognostic judgements as well as personal judgements about the ability to treat the patients. | Health status bias; Sexual orientation bias (Stereotype bias) | Y |
| Batson & Marz, 1979; USA^6^ | Journal Article | Psychology | Clinical psychology graduate students; general psychology students (n = 28) | Participants were separated into two groups: one trained professionals and one untrained non-professionals group. All participants took part in intake interviews with two clients (confederates) who presented a problem that was either relatively dispositional or relatively situational. Participants were asked to complete two diagnostic questions on an 8-point Likert scale. | Diagnosis | Predicted that trained professionals would be more dispositional in their diagnostic assessments of the clients compared with untrained non-professionals, and that trained professionals would not discriminate between the two types of client problem in their diagnostic assessments. | Dispositional bias | Y |
| Beasley & Manning, 1973; USA^7^ | Journal Article | Speech Pathology | Speech pathology language evaluators (n = 40) | Participants received one of four types of pre-information: negative; positive; incomplete; no pre-information. They then evaluated tape-recorded language samples using three objective measures and four subjective measures to assess language performance. | Assessment | Predicted that the four types of pre-information would produce variance in participants’ language performance assessments. | Experimenter bias | N |
| Becker, 1994; USA^8^ | Journal Article | Psychology; Social Work | Clinical psychologists; Clinical social workers; psychiatrists (n = 311) | Participants were randomly assigned to one of six case vignettes, each including a history of sexual abuse. Two versions of three separate cases each described criteria that met a diagnosis of bipolar disorder or post-traumatic stress disorder, and depicted either a female or male character. Participants were asked to rate the extent to which the case characters appeared to have multiple Axis 1 or II disorders. | Diagnosis | Predicted that participants would more frequently apply a bipolar disorder diagnosis to women and post-traumatic stress disorder to men. | Sex bias | Y |
| Benefee et al., 1976; USA^9^ | Journal Article | Psychology; Social Work | Mental health practitioners, including: psychologists; social workers; nurses; psychiatrists; education (n = 55) | Participants, who all identified as black, read one of two versions of a case profile vignette depicting either a white or black character. Participants were asked to provide clinical impressions on a series of 7-point Likert scales, and also completed a traditional social beliefs scale. | Diagnosis | Predicted that participants’ clinical impressions would be more favourable when the case character was depicted as black, and when participants were scored as less traditional on the traditional social beliefs scale. | Racial bias | Y |
| Benjamin, 2017; USA^10^ | Psy.D. Dissertation | Psychology | Masters and Advanced Clinical Psychology graduate students; Licensed clinical/counselling psychologists (n = 152) | Participants were randomly assigned to one of two case vignettes, which differed only by the racial identity of the vignette character, either Caucasian or African American. Participants were asked to assign one or more diagnoses from a list of 10, or no diagnosis. | Diagnosis | Increased likelihood of a diagnosis of Antisocial Personality Disorder was predicted for the case study describing an African American male. | Racial Bias | N |
| Bernstein & Lecomte, 1982; Canada^11^ | Journal Article | Psychology; Social Work | Counsellors; clinical social workers; clinical psychologists (in training and certified) (n = 893) | Participants were separated into groups by gender, profession and training level. Participants within each group received one of two versions of a case vignette describing the same client, but portrayed as either female or male. Participants were asked to complete a therapist expectancies inventory to determine participants’ pre-counselling diagnostic expectancies (anticipation of client need) and prognostic judgements. | Prognosis | Predicted that participant gender, profession and/or training level may alter participants’ diagnostic expectancies and prognostic judgements, and that these would also differ depending on the case characters’ gender. | Gender bias (sex bias) | N |
| Bieri et al., 1963; USA^12^ | Journal Article | Social Work | Graduate social work students (n = 176) | Participants read three cases, varying in the described pathology of the client (‘extreme’ case, ‘middle’ case, ‘low’ case). Case pathology level and the type of pathology described was systematically ordered (dependency/aggression), such that eight experimental groups read the cases in a different sequential order. Extreme cases were designed to produce a high anchor and middle cases a low anchor. Participants then assessed the focus case on a graphic 20-point pathology scale, from "very mild maladjustment" to "extreme maladjustment." | Assessment | Predicted that pathology scale assessments for the middle focus case would be significantly different when a participant read high anchor cases compared to when participants read low anchor cases earlier in the case sequence. Also made additional predictions for the degree and direction of these differences. | Anchoring effects | Y |
| Billingsley, 1977; USA^13^ | Journal Article | Psychology; Social Work | Psychologists; psychiatrists; psychiatric social workers (n = 64) | Participants were grouped by their sex, and read one of two versions of two case vignettes (depicting an ‘explosive’ or ‘restricted’ client problem) that differed by the sex of the case character (male or female). Participants were asked to respond to questions relating to diagnosis, prognosis, and treatment intentions. | Diagnosis; Prognosis; Treatment | Predicted that treatment intentions for female characters would emphasize stereotypically female behaviours and treatment intentions for male characters would emphasise stereotypically male behaviours, regardless of client pathology. | Sex bias | N |
| Bloch et al., 1980; USA^14^ | Journal Article | Psychology | Mental health professionals (n = 34) | Participants, who all identified as ‘white’, read one of two versions of a case profile vignette depicting either a ‘white’ or ‘black’ character. Participants were asked to provide clinical impressions on a series of 7-point Likert scales relating to assessment and therapeutic treatment prognosis. | Assessment; Prognosis | Expected that racial attribution of the case character would elicit differences in assessment scores and therapeutic reatment prognosis. | Racial bias | N |
| Brailey & Vasterling, 2001; USA, Canada^15^ | Journal Article | Psychology | Clinical Psychology graduate students; Licensed clinical psychologists (n = 40) | Participants were grouped into two experimental groups, based on having low and high psychodiagnostic experience. Participants were provided with diagnostic information, watched a videotaped client interview and underwent three separate memory tasks. | Diagnosis | Memory errors: High experience clinicians would demonstrate enhanced memory for relevant information. Coherence bias: Predicted that high experience clinicians would use more personal criteria to create coherence. Framing effect: Predicted that low experience clinicians would provide more criteria to support a primary diagnosis, rather than an alternative diagnosis. Decision-consistent bias: Predicted that low experience clinicians would provide more criteria for a diagnosis labelled correct. Confirmation bias: High experience clinicians predicted to use more disconfirming information. | Memory errors; Coherence bias; Framing effect; Decision-consistent bias; Confirmation bias | Y |
| Bresler, 1984; USA^16^ | PhD Dissertation | Psychology | Clinical psychologists; Undergraduate students (n = 652) | Participants were asked to answer three questions relating to six vignettes. Two vignettes depicted male role-related pathology, two described female role-related pathology, and two were "noise" cases, describing non-sex-role related problem behaviours. Participants responded to one of eight targets: female or male; child, adolescent, young adult, old adult were varied across the vignettes. Participants also completed a Personal Attribute Questionnaire. | Assessment; Prognosis; Treatment | Predicted that participants assessment, prognosis and treatment responses would be influenced by the sex and age of the vignette character, as well as the sex-role-related pathology of targets. Also, predicted that mental health standards would alter with age and sex. | Age bias; Sex bias; Sex role stereotype bias | Y |
| BruchmÜller & Schneider, 2012; Germany^17^ | Journal Article | Psychology; Social Work | Psychologists; Psychiatrists; Social workers, all specialising in children and adolescents (n = 463) | Four vignettes described cases with varying criteria meeting a diagnosis for Attention Deficit and Hyperactivity Disorder (ADHD). All four vignettes had boy and girl versions with no other change in the information provided these were assigned randomly to participants. Participants were asked to provide a diagnosis and treatment recommendation. | Diagnosis; Treatment | Representativeness heuristic: Predicted that practitioners would overdiagnose ADHD if the case represents a prototypical ADHD case presentation even when not meeting diagnostic standards. Sex bias: Vignettes with male characters predicted to be diagnosed with ADHD more often. Overdiagnosis predicted to lead to treatment decisions. | Representativeness heuristic; Sex bias | Y |
| Castriano Galante, 1999; USA^18^ | PhD Dissertation | Social Work | Child welfare social workers (n = 907) | The study included two case vignettes describing either a physical abuse case or a child neglect case. Vignettes were varied by case ambiguity and risk (low ambiguity/low risk of future harm; low ambiguity/high risk of future harm; high ambiguity/moderate risk of future harm); and race of child (Caucasian; African American). Participants were asked to read one vignette variation, and were asked to respond to questions relating to service provision for the vignette family. | Assessment; Treatment; Child placement | Multiple participant responses were analysed against the case vignette conditions: maltreatment type; ambiguity level; child race. Predicted that decision making patterns would differ by maltreatment type and race of the child. | Racial bias; Bias relating to type of abuse | Y |
| Commisso, 1984; USA^19^ | PhD Dissertation | Psychology; Social Work | Counsellors; Psychologists; Social workers; Psychiatrists; Psychiatric nurses and other training backgrounds (n = 133) | Participants were assigned four vignette conditions, within each of which the same information was presented, but in a different order. Participants were asked to assess the character’s functioning, and align this with a diagnosis and prognosis, and were also asked to rate their own confidence in their decision. | Assessment; Diagnosis; Prognosis | Anchoring and adjustment: The order of information presented was predicted to influence the ratings of client functioning, diagnosis and prognosis, and participants were expected to adjust their judgements from the initial anchor as they received new information. Confirmation bias: Would have been indicated by a lack of adjustment from participants initial impression. | Anchoring and adjustment; Confirmation bias | N |
| Curtis, 2002; USA^20^ | PhD Dissertation | Psychology; Social Work | Clinical, School and Counselling Psychologists; Social Workers (n = 88) | Participants each received two case vignettes, one critical case depicting an adolescent with conduct disorder and one distractor case depicting an adolescent with schizophrenia. A male and female version of each vignette existed, and these were randomly distributed to participants. Participants were asked to provide a diagnosis, and a global assessment of functioning, including severity of symptoms ratings and prognosis. | Diagnosis; Prognosis; Assessment | Current and expected score on the Global Assessment of Functioning scale were used to assess current assessment of functioning and prognosis. It was predicted that participants would diagnose conduct disorder more for male vignettes than for females, and that current functioning and prognosis would differ between female and male vignettes. | Gender bias (sex bias) | Y |
| Crosby & Sprock, 2004; USA^21^ | Journal Article | Psychology | Psychologists (n = 167) | Participants were asked to read two case vignettes: one portraying a hypothetical patient with antisocial personality disorder, and the other portraying an unrelated disorder that was intended to divert participants from the purpose of the study. Identical male and female versions of the cases were developed. Participants were asked to read one version of the each vignette and complete a diagnostic questionnaire that included: symptom ratings, diagnostic ratings, and a diagnosis. They were then asked to choose a diagnosis from provided options that was most representative of the case, and rate their confidence in the diagnosis. | Assessment; Diagnosis; Prognosis | Predicted that when patient sex was consistent with the gender weightings for a disorder (male, antisocial) the target diagnosis would be assigned more frequently and its diagnostic criteria would be rated as more representative, participants would report higher diagnostic confidence, severity of symptomology would be rated lower and prognosis rated higher. | Sex bias; Sex role stereotype bias; Consistency effect (measuring overconfidence) | Y |
| Cwik et al., 2016; Germany^22^ | Journal Article | Psychology | Psychologists (n = 475) | Each participant assessed three case vignettes that portrayed clients fulfilling diagnostic criteria for either major depressive disorder, generalised anxiety disorder or borderline personality disorder. Participants were randomly assigned to groups in a 2 (diagnostic method: with or without diagnostic checklist) x 2 (gender: male vs female case vignette) between subjects design. Participants were asked to complete a questionnaire that included items relating to diagnosis and treatment. | Diagnosis; Treatment | Predicted that there would be no association between gender and either diagnostic accuracy or treatment recommendations. | Sex bias | N |
| Dailley, 1980; USA^23^ | Journal Article | Social Work | Social workers (n = 207) | Participants were asked to read one of four versions of the same clinical vignette in which sex (male, female) and personality type (passive, aggressive) were varied. Participants were asked to complete a clinical judgement inventory. | Assessment; Prognosis | Predicted that both sex and sex role would alter clinical judgement, including prognosis. | Sex bias; Sex role stereotype bias | Y |
| Dailley, 1983; USA^24^ | Journal Article | Social Work | Social workers (n = 328) | Participants were asked to assess one of three versions of the same case vignette in which the client displayed personality characteristics that were either aggressive, passive, or both aggressive and passive. Vignette characters were also altered to be either female, male or androgynous. Participants were asked to complete a clinical judgement inventory and a sex role inventory. | Assessment | Predicted that clinical judgements would differ with the sex role of the client, irrespective of biological sex. | Sex role stereotype bias | Y |
| Delphin, 2001; USA^25^ | PhD Dissertation | Psychology | Psychologists specialising in personality assessment (n = 137) | Two case vignettes depicting borderline personality disorder (BPD) and antisocial personality disorder (ASPD) were modified such that the case characters were either female or male, African American or European American. Half of the participants received either African American or European American vignettes and half of these participants received vignettes depicting either females or male characters. Participants were asked to diagnose the case character. | Diagnosis | Predicted that for the separate vignettes: African American males would receive an ASPD diagnosis more than other groups; European males would receive a BPD diagnosis less than other groups; African American females would receive a BPD diagnosis more than other groups. Across vignettes, it was predicted: African Americans would receive more diagnoses than European Americans; level of impairment would be perceived less for European Americans that for African Americans; more females than males would receive a BPD diagnosis; more males than females would receive an ASPD diagnosis. | Gender bias (sex bias); Ethnic bias (racial bias); Overconfidence | Y |
| Dewhurst, 2007; USA^26^ | Journal article | Genetic Counselling | Genetic counselling students; Genetic counsellors (n = 249) | Participants received four genetic problems that assessed their use of probability rules and the conjunction effect. Student and genetic counsellors’ results were compared. | Genetic likelihood problems | Predicted that participants would answer problems incorrectly which would indicate the misuse of probability rules. Predicted differences between more experienced genetic counsellors and counselling students. | Misuse of probability rules; Conjunction effect (representativeness heuristic); | Y |
| Dikert, 1988; USA^27^ | Journal Article | Social Work; Psychology | Mental health professionals (n = 80) | Participants were asked to evaluate four case descriptions that differed in type and degree of symptoms. The hearing status of the case character was presented to participants as either deaf or not hearing impaired. Participants were asked to undertake a psychiatric evaluation that included mental health ratings, and recommendations for the level of chemotherapy and level of supervisory care. Participants’ attitudes towards the hearing impaired were also assessed. | Assessment; Treatment | Predicted that participants would be more likely to attribute the dysfunction to deafness for deaf cases, rather than to mental illness. It was also predicted that more restrictive forms of psychiatric treatment would be recommended for deaf patients, with higher levels of chemotherapy and supervision, compared to hearing patients. | Bias against hearing impairment (diagnostic overshadowing) | Y |
| DiNardo, 1975; USA^28^ | Journal Article | Psychology | Graduate psychology students (n = 60) | Participants were randomly assigned to one of six conditions. The same case history and videotaped interview, depicting a healthy man, was adapted for social class (lower and middle) and diagnostic suggestion (no suggestion/ psychologist suggestion/ psychiatrist suggestion). Participants were then asked to choose one of four diagnostic categories and a prognosis on a 9-point scale. | Diagnosis; Prognosis | Predicted that social class and diagnostic suggestion would influence the diagnostic and prognostic assessments made by participants. | Socioeconomic bias; Expectancy effect (Diagnostic suggestion) | Y |
| Diver, 1998; UK^29^ | Doctor of Clinical Psychology Thesis | Psychology | Clinical psychologists (n = 36) | Participants were randomly assigned to experimental and control group. All participants were asked to read a case transcript, divided into three parts, and answer questions. Participants in the experimental group were asked to keep notes as they read the transcript and to list any evidence that was inconsistent with their two main hypotheses for the case (potential debiasing activities). | Diagnosis | Participant answers to the follow up questions were used to represent a range of variables, including confidence and accuracy. Confidence was predicted to be lower for the experimental group compared to the control, and more closely aligned with accuracy for the experimental group. | Overconfidence | N |
| Doyle, 1987; Australia^30^ | Journal Article | Audiology | Audiologists (n = 54) | Participants rated the intelligibility of taped samples of hearing-impaired speech (poor/good), and were randomised to different conditions of accompanying audiogram labelling information (no label/mild-moderate/profound). Participants were then asked to complete a speech intelligibility rating scale. | Assessment | Predicted that the audiogram label would alter participants’ intelligibility ratings (a stereotyped expectation of the speech intelligibility of children with particular degrees of hearing loss). | Stereotyping (Speech intelligibility) | Y |
| Eadie et al., 2011; USA^31^ | Journal Article | Speech Pathology | Speech-language pathologists; novice listeners (n = 28) | Twenty-six speakers with dysphonia and four normal controls provided speech recordings. Novice and experienced clinicians evaluated speech samples for roughness and breathiness using 100-mm visual analogue scales. In one condition, the speech samples were presented without diagnostic information; in the second condition, samples were presented in conjunction with the medical diagnosis. | Assessment | Predicted that participant judgements would be more severe when they were given prior information about the diagnosis, and that this may be mediated by clinician experience. | Expectancy effect (Prior knowledge of medical diagnosis) | Y |
| Egeland, 1969; USA^32^ | Journal Article | Psychology | Graduate students in education and psychology n = 46) | Participants scored responses to the Wechsler Intelligence Scale for Children (WISC). They were assigned of three conditions relating to the information they received about past academic and intellectual level of the focus child: slow learner; above-average; no expectancy information. | Assessment | Predicted that the expectancy information would influence the scoring of the WISC by participants. | Examiner bias | Y |
| Enosh & Bayer-Topilsky, 2014; Israel^33^ | Journal Article | Social Work | Child welfare social workers (n = 105) | Participants were asked to respond to eight vignettes, each followed by two questions; an assessment of risk, and recommendation of out of home care. Vignettes were manipulated for objective level of risk (low, high, ambiguous), socio-economic level of family (moderate-high, low), and ethnicity (Mizrahi, Ashkenazy). Version were randomly assigned to participants. | Assessment; Child placement | Predicted that for case vignettes depicting ambiguous levels of risk, socioeconomic and racial bias would influence participants’ judgements such that the socioeconomic and racial status of families would predict increased recommendations for out of home care. | Socioeconomic status bias; Racial bias | Y |
| Fantasia-Davis, 1997; USA^34^ | PhD Dissertation | Psychology | Psychologists (n = 348) | Two vignettes were used; each described a case with symptoms of psychological disturbance meeting criteria for DSM V diagnosis of Depression, Single Episode, or symptomology for a diagnosis of Borderline Personality Disorder. Participants were randomly assigned to one of six conditions: major depression, single episode/diagnosis-specific treatment base rate; major depression, single episode/general treatment base rate; major depression, single episode/no treatment base rate; borderline personality disorder/diagnosis specific base rate; borderline personality disorder/general base rate; borderline personality disorder/no base rate. Participants were asked to rate the likelihood that the assigned case would show measurable improvement within a given range of outpatient psychotherapy sessions. | Prognosis | It was predicted that quantitative base rate information on number of sessions required for the alleviation of symptoms, provided in the case vignettes, would influence a clinical probability judgement on treatment outcome. | Base rate fallacy | N |
| Fernandez, 2012; USA^35^ | Degree in Clinical Psychology Thesis | Psychology | Doctoral level clinical psychologists (n = 111) | Participants read one of two versions of a vignette outlining a case containing a mixture of psychotic or mood disorder features. The two versions varied only by the character’s race (African American/Caucasian). Participants were asked to rate symptomology and make a diagnosis, using one of two diagnostic approaches. | Diagnosis | It was predicted that cases depicting the African American character would receive comparably more schizophrenia diagnoses that the vignette depicting a Caucasian character, particularly when a ‘prototype’ diagnostic approach was used. | Racial bias | N |
| Fernbach, 1974; USA^36^ | Doctor of Psychology Thesis | Psychology | Licensed clinical psychologists (n = 119) | Each participant read one version of two case vignettes depicting either antisocial personality disorder or somatization disorder. Vignettes varies by patient sex (female, male). Participants were asked to choose a diagnosis, treatment recommendation, assess prognosis and recommend length of treatment. | Diagnosis; Treatment; Prognosis | It was predicted that participants would be more less likely to diagnose antisocial personality disorder for females than for males, and more likely to diagnose somatisation disorder for females than for males. It was also predicted that the vignette characters’ sex would influence treatment recommendations, assessments of prognosis and length of treatment recommendations. | Sex bias | Y |
| Fernbach et al., 1989; USA^37^ | Journal Article | As above |  |  |  |  |  |  |
| Feinblatt & Gold, 1976; USA^38^ | Journal Article | Psychology | Graduate students of clinical or school psychology (n = 27) (Phase II, Psychologists Sample) | Participants responded to one of four versions of a case description depicting a nine-year-old presenting with behaviour problems. Two versions described the child as emotional and withdrawn (female stereotype) and two versions described the child as aggressive and defiant (male stereotype). Sex (boy/girl) was also varied across the versions. Participants rated the case for severity of the problem, made treatment recommendations and were asked about their prediction for future success of the child if their behaviour continued. | Assessment; Treatment; Prognosis | Predicted that the severity scores, treatment recommendations and predictions of future success would differ with the sex and stereotypical sex role behaviour depicted in the case scenario, such that when sex and sex role behaviour was not congruent, severity scores would increase, recommendations for treatment increase, and predictions for future success would be lower. | Sex bias; Sex role stereotype bias | Y |
| Fischer & Miller, 1973; USA^39^ | Journal Article | Social Work | Social workers (n = 360) | Participants read one version of two case histories one mild, one severe psychopathology described), in which the case character race (‘white’, ‘black’) and socioeconomic status (lower, upper) varied. Participants were asked to complete a treatment inventory and demographic questionnaire. | Assessment; Treatment | Predicted that when the client was depicted as lower class and/or as ‘black’, participants would make a less favourable assessment. | Racial bias; Socioeconomic status bias | Y |
| Fischer et al., 1976; USA^40^ | Journal Article | Social Work | Social workers (n = 135) | Participants were asked to read one of four versions of the same clinical vignette where sex (male, female) and personality type (passive, aggressive) were varied. Participants were asked to complete a clinical judgement inventory. | Assessment; Prognosis | Predicted that both sex and sex role would alter clinical judgement, including prognosis. | Sex bias; Sex role stereotype bias | Y |
| Ford & Widiger, 1989; USA^41^ | Journal Article | Psychology | Psychologists (n = 354) | Participants were presented with one of three versions (male, female, sex unspecified) of three case vignettes (ambiguous, histrionic, antisocial), or one of three possible lists of individual behaviours (reflecting the case histories). Participants rated the likelihood that the case character/list reflected a list of disorders. | Diagnosis | Predicted that diagnosis would be influenced by the sex of the vignette character. Predicted that if base rates of disorders appropriately influenced diagnosis, then when a case is more ambiguous, the effect of sex would be more pronounced than for cases where the diagnostic criteria are clearly met. | Sex bias | Y |
| Frame et al., 1982; USA^42^ | Journal Article | Psychology | School psychologists (n = 24) | A hypothetical case was accompanied by a referral form that contained systematic variations in the focus child’s race (‘white’/’black’), socioeconomic status (custodian/physician) and school achievement levels (low/high). Participants assessed the simulated case using diagnostic and treatment measures. | Diagnosis; Treatment | Predicted that race, socioeconomic status and school achievement levels would produce variance in participants’ diagnostic assessment and treatment recommendations. | Racial bias; Socioeconomic status bias; | Y |
| Franklin & Grossman, 1990; USA^43^ | Journal Article | Speech Pathology | Certified speech-language pathologists (n = 111) | Participants completed a survey which included information about the referral and assessment of a hypothetical case study child. Only the sex (female/male) and the parents’ occupations (to reflect socioeconomic status) were varied. Participants were asked to respond to a set of statements about the child along a six-point scale. | Assessment; Referral; School placement | Predicted that participant responses to the set of statements relating to assessment, referral and placement decisions would differ by sex of the child and by parent occupation (socioeconomic status proxy). | Sex bias; Socioeconomic bias | Y |
| Friedlander & Stockman, 1983; USA^44^ | Journal Article | Psychology; Social Work | Psychologists; psychiatrists; social workers (n = 46) | Participants were presented with one of two versions of two cases (presented as five separate interview summaries) that varied in the placement of pathognomonic information (early, late). Participants were also randomised to a publicity condition, where they were asked, or not asked, to note the salient information that led to their clinical judgement. All participants were asked to provide an assessment of functioning and prognosis. | Assessment; Prognosis | Anchoring: Predicted that the time of exposure to pathognomonic information would influence assessment of functioning and prognosis, such that those who received pathognomonic information earlier would have lower estimates of functioning and prognosis. Publicity effect: Predicted that more conservative estimates of functioning and prognosis would be observed for participants who were asked to justify their clinical judgement. | Anchoring; Publicity effect | Y |
| Gale et al., 2016; UK^45^ | Journal article | Social Work | Mental health professionals, including medical doctors, nurses, social workers (n = 400) | Participants were purposively distributed to four conditions, each viewing the same vignette depicting a young male with severe and enduring mental illness. The vignette was presented with no image of the client, or an image of the client: smiling; moderately sad; moderately angry. Participants were asked to rate the risk of suicide and provide a rating of their own confidence. | Assessment | It was predicted that participants who received the sad face would rate the client as more likely to commit suicide than those who received the vignette with no picture, a happy, or angry face. The vignette was framed to give equal probability (50%) to no suicide and suicide outcomes. An overall deviation from equal numbers of participants assessing a suicide risk and no suicide risk, was reported as a biased assessment of risk. Given the vignette framing, any rating of confidence over ‘not confidence’ was deemed as a biased overconfident response. | Availability bias; Overconfidence bias | Y |
| Gammon, 2000; USA^46^ | PhD Dissertation | Social Work | Social workers (n = 534) | Four case vignettes were developed, with variations in the racial background (African American/Caucasian) and socioeconomic status (low/middle) of the case characters. Participants were randomly allocated one of these variations. Participants were asked to make an assessment about where the case child, who had been in foster care for 17-months, should be reunified with family. | Child placement | It was predicted that for vignettes that predicted the child as African American and vignettes that depicted the family as from a low socioeconomic background, participants would be less likely to recommend reunifying the family. | Racial bias; Socioeconomic bias | N |
| Garb, 1996; USA^47^ | Journal Article | Psychology | Psychologists (n = 59 (Study 2) | Participants read a case history of a man who may meet the criteria for a diagnosis of schizophrenia. Participants at two medical centres were told that the patient  described by the case history was ‘black’ and participants at three other medical centres were told that the patient was ‘white’. Participants were then asked to rate the likelihood of the person's having schizophrenia, the likelihood of major  depression, and the likelihood of brief reactive psychosis. | Assessment; Diagnosis | It was predicted that when participants were told that the case character was ‘black’, the likelihood ratings for each of the disorders would be higher.  (*Note:* The primary aim of the three studies are presented in this publication was to ascertain whether clinicians used the representativeness heuristic, not to detect bias *per se*) | Racial bias | N |
| Garfield et al., 1973; USA^48^ | Journal Article | Psychology | Elementary and special counsellors (n = 18) | Participants randomly allocated to one of two groups. Participants read an identical case history with social history information that varied; one reflecting an upper-middle class social position and the other reflecting a lower-class status. Participants were asked to complete a questionnaire assessing course of action and likely outcomes. | Treatment; Prognosis | Predicted that responses for the course of action and likely outcomes would differ for participants in the upper-middle class and lower class conditions. | Socioeconomic status bias | Y |
| Garner et al., 1994; USA^49^ | Journal Article | Psychology | Rehabilitation counsellors or related occupation (n = 89) | Participants read one of five variations of the same case description, describing a person with no diagnosed disability; a traumatic brain injury; a hearing impairment; epilepsy; an IQ of 65. The versions differed only in the specific disability condition described and described features of a thought disorder. Participants responded to a questionnaire that assessed diagnostic impressions and treatment recommendations. | Diagnosis; Treatment | Predicted that participants may exhibit diagnostic overshadowing bias, depicted by changes in their diagnostic or treatment decisions, when the case description included a client with a mental or physical disability, compared to when a case depicted a person with no disability. | Diagnostic overshadowing bias | Y |
| Goldsmith & Schloss, 1984; USA^50^ | Journal Article | Psychology | School psychologists | Two patient variables (stereotypical sex role and sex) and two participant variables (more or less traditional sex role and sex) were manipulated. | Diagnosis; Treatment |  | Diagnostic overshadowing | Y |
| Gomes & Ambramowitz, 1976; USA^51^ | Journal Article | Psychology | Psychotherapists (n = 182) | Participants read one version of a case history differing only with respect to sex and a concluding paragraph within which a cluster of stereotypical feminine or masculine traits was outlined. Participants were asked to complete a brief evaluation form including diagnostic and prognostic judgements. | Diagnosis; Prognosis | Predicted that sex, and sex role characteristics of both the vignette character and the participants would influence a range of clinical judgements. | Sex bias; Sex role stereotype bias | N |
| Gordon, 2010; USA^52^ | PhD Dissertation | Psychology | Psychologists n = 135) | Participants were presented with one of eight versions of an identical case vignette that differed only in the client sex (male, female), sexual orientation (heterosexual, lesbian/gay) and gender role (feminine, masculine). Participants were asked to complete measures assessing diagnostic impressions, the attractiveness of the client, and their own level of heterosexual identity development. | Diagnosis | Predicted that clinical decisions will differ as a result of client sex, sexual orientation and client gender role when participant heterosexual identity development status is controlled for. | Sex bias; Sex role stereotype bias; Sexual orientation bias | Y |
| Gross, 2015; USA^53^ | PhD Dissertation | Psychology | Doctoral level psychology students (n = 38) | Four versions of the same vignette varied in the depicted client’s occupation (no occupations; non-artistic occupation; visual arts occupation; performing arts occupation). Participants assessed one version of the vignettes, and were asked to provide a brief clinical and diagnostic impression of the client, and complete a diagnostic impressions questionnaire. | Diagnosis | It was predicted that participants would assume less perceived psychopathology for clients who were not depicted as having an arts-based vocation. | Vocation bias | N |
| Grupp & Glass, 1980; USA^54^ | Journal Article | Speech Pathology | Speech-language pathologists (n = 40) | Participants read two forms of a questionnaire where a child with an articulation disorder was depicted alongside a set of ‘female behaviours’ or ‘male behaviours’ and given a stereotypical male or female name in the different versions. Participants were asked to answer a single question related to whether they would alter the child’s behaviour. | Assessment | Predicted that when the behaviour depicted did not conform to sexual stereotypes, then participants would more likely recommend that the behaviour be modified. | Sex bias; Sex role stereotype bias | Y |
| Haase, 1956; USA^55^ | PhD Dissertation | Psychology | Psychologists (n = 75) | Participants were presented with four pairs of Rorschach reports that varied by socioeconomic status of the client outlined within a case history. Participants were asked to evaluate the records on assessment, diagnosis and prognosis. | Assessment; Diagnosis; Prognosis | Participants would assess greater pathology and less favourable prognoses to protocols depicted as lower class. Also predicted that several participant measures would mediate this effect. | Socioeconomic status bias | Y |
| Hamilton et al., 1986; USA^56^ | Journal Article | Psychology | Clinical psychologists (n = 65) | Participants independently diagnosed 18 case study vignettes on the basis of 10 possible diagnostic categories, including antisocial and histrionic personality. The  l0 critical profiles described one male and one female representing one of five levels of pathology: (a) all antisocial behaviour, (b) all histrionic characteristics, (c) a predominance of antisocial behaviours with histrionic symptoms, (d) a predominance of histrionic descriptors with antisocial symptoms, or (e) an equal combination of histrionic and antisocial indicators. The remaining case studies presented typical descriptions of eight other disorders not commonly seen in either sex. | Diagnosis | Predicted that participants would be more likely to assign a histrionic personality diagnosis to female clients than male clients showing identical symptoms. It was also predicted that sex bias would be more pronounced for opposite sex participants and for vignettes that described fewer diagnostic symptomology. | Sex bias | Y |
| Hansen, 2015; USA^57^ | PhD Dissertation | Psychology | School psychologist students and alumni (n = 81) | Each participant was randomly assigned to one of four groups. Three experimental groups received one of three vignettes described as a teacher’s report of the target student, and contained symptoms relating to one of three disorders: Emotional Disturbance, Attention Deficit Hyperactivity Disorder or Learning Disability. A teacher report was not given to the control group. Participants were then asked to assess the same video of the target child. | Assessment | Predicted that school psychologist participants in the control group would undertake systematic observations accurately, and that information gathered during the referral process given to given to the experimental groups would bias the observational assessments. | Labelling bias | N |
| Hardy & Johnson, 1992; USA^58^ | Journal Article | Psychology | Graduate clinical and counselling students (n = 185) | Participants assessed one version of a case vignette in which client sex, alcoholic status and socioeconomic status was manipulated. Participants were asked to complete measures relating to several prognostic and treatment variables. | Prognosis; Treatment | Predicted that sex (female), socioeconomic status and alcoholic status would result in more negative prognostic and treatment evaluations of the vignette client character. | Sex bias; Socioeconomic status bias; Mental health bias | Y |
| Haverkamp, 1993; USA^59^ | Journal Article | Psychology | Graduate counselling psychologists (n = 66) | Participants were randomly assigned to one of two videotape conditions, and were asked to watch one of two versions of a client/counsellor interview that differed only in the client’s description of their own problem. Participants undertook a number of tasks relating to identification of the problem (self-generated hypothesis), verbal and non-verbal client cues, client descriptors, counselling behaviour and treatment actions. | Assessment; Treatment | Predicted that participants’ responses to the five tasks would be more confirmatory in relation of their own self-generated hypothesis about the client, as oppose to being neutral or disconfirmatory. | Confirmation bias | Y |
| Hieger, 2007; USA^60^ | PhD Dissertation | Psychology | Graduate counselling psychologists; trained counselling psychologists (n = 66) | Participants read one of four versions of a clinical vignette which differed only in: diagnostic ambiguity (clear, ambiguous) and race of the client (‘white’, African American). Race of the participants was included as an independent variable. Participants completed questionnaires related to assessment and treatment planning and prognostic outlook, and also completed a discrimination measure. | Assessment; Treatment; Prognosis | Predicted that participants would rate symptom severity higher and prognosis outlook lower when the race of the case character was dissimilar to their own, when the case was ambiguous and when levels of reported prejudice were higher. | Racial bias | N |
| Hersh, 1971; USA^61^ | Journal Article | Psychology | Graduate student psychologists (n = 28) | Participants (testers) administered and interpreted Stanford-Binet Intelligence Scale to participating children. Participants were randomised to receive a referral letter that contained either positive or negative information about the child. Male and female participants were also randomised to assess either a male or female child. Thus, seven male and seven female testers each tested a positively referred male and a negatively referred male, and another seven male and seven female testers who each tested a positively referred female and a negatively referred female. | Assessment | Predicted that participants would perform differently when receiving positive teacher referral reports than when receiving negative teacher referrals for intellectual evaluation. Predicted that they would obtain a significantly higher IQ score for the positive than the negative referral; give more warmth to the positively referred children than to the negative; rate the positively referred children's behaviour during the test as more favourable, and; make more favourable recommendations for the positively referred children than for the negatively referred. | Expectancy effect | Y |
| Howell, 2009; USA^62^ | PhD Dissertation | Social Work | Child protection intake workers (n = 87)­­ | Participants were randomly assigned to a series of 24 child maltreatment vignettes where the race and drug use of characters was manipulated. Participants were then asked to complete a number of questions relating to child protection intake (accept/reject) and complete Race and Drug subscales. | Assessment | It was predicted that: cases involving Caucasian children would be accepted less frequently; participants with higher scores on the Drug Subscale, would accept more cases for investigation and choose more decision factors relating to drug use than participants with lower scores. | Racial bias; Drug use bias | Y |
| Howells Wrobel, 1993; USA^63^ | Journal Article | Psychology | Clinical psychologists (n = 209) | Participants read one version of a clinical vignette describing a patient with cognitive and affective symptoms common in depressed patients. Ten versions of the vignette existed, allowing for variation in sex (female, male) and five age levels (45-85, at 10 year intervals). Participants responded to questions related to: diagnosis; treatment setting; treatment, and; prognosis. | Diagnosis; Treatment; Prognosis | Predicted that patient age and sex would result in differences in participants’ clinical decisions regarding diagnosis (taking into account base rate data), treatment and prognosis. Also predicted that clinician would use different information portrayed in the case vignettes to make clinical decisions, relying on stereotyping rather than base rate data. | Age bias; Sex bias | Y |
| Hueber & Cummings, 1985; USA^64^ | Journal Article | Psychology | School psychologists (n = 56) | Participants reviewed a brief case description of a child referred because of learning problems. Participants then undertook a simulated education assessment of the child. Participants were randomly placed in one of four treatment conditions varying the child’s sociocultural background (rural vs. suburban) in the case study description and the nature of the assessment results (normal vs. learning-disabled). | Assessment; Diagnosis; School Placement | Predicted that knowledge of a student’s sociocultural background and/or the nature of the individual assessment data would influence: expectations regarding students’  future competence in academic and social areas; diagnostic decisions,  and; educational placement decisions. | Socioeconomic status bias | N |
| Huebner, 1990; USA^65^ | Journal article | Psychology | Psychologists (n = 88) | A case vignette described a young male student alongside medical and intelligence scores, behaviour and referral information. Four versions were assigned randomly to participants which differed in the prior educational placement of the case child (special education resource placements vs no placement) and the current test results (learning difficulty vs normal range). Participants were asked to make diagnostic and school placement decisions. | Diagnosis; School placement | It as predicted that where the case character had been previously assigned to a special education program, this would influence participants’ diagnostic and school placement decisions, with an increased likelihood of a learning difficulty diagnosis and special education placement. | Confirmation bias | N |
| Jacob, 2010; USA^66^ | PhD Dissertation | Psychology; Social Work | Counsellors; Psychologists; Social workers (n = 182) | Two versions of a case vignette, with and without additional contextual information, were assigned randomly to participants who were asked to recommend either individual or couples counselling. Participants completed measures to assess inherent gender-related biases and emotional contagion. | Treatment | Emotional contagion: Higher Emotional Contagion Scale (ECS) score were predicted to be associated with recommendations for couples counselling. Gender-related biases: Higher scores on the Beliefs About Men’s Emotions scale (BAME) were predicted to be associated with recommendations for individual counselling. | Emotional contagion; Gender-related biases (sex bias) | N |
| James & Haley, 1995; USA^67^ | Journal Article | Psychology | Clinical psychologists (n = 371) | Participants responded to one version of a vignette that was manipulated for age (35, 70) and health (unremarkable or poor). Participants responded to a range of questions relating to clinical and personal judgements of the client, including diagnosis, treatment and prognosis. | Diagnosis; Treatment; Prognosis | Predicted that the age of the case character would have some effect on clinical judgements, and that age combined with health status would have the most influence on clinical judgements | Age bias; Health status bias | Y |
| Jenkins-Hall & Sacco, 1991; USA^68^ | Journal Articles | Psychology | Psychotherapists (n = 62) | Participants viewed one version of a scripted videotape interaction between a client and a therapist. The client was depicted as either ‘black’ and depressed, ‘white’ and depressed, ‘black’ and not depressed, or ‘white’ and not depressed. Participants were asked to evaluate the client using a range of measures relating to diagnostic and personal assessment | Assessment | Predicted that participants would make more negative evaluations when the client was depicted as ‘black’ and/or depressed. | Racial bias; Mental health bias | Y |
| Jenkins, 2012; USA^69^ | PhD Dissertation | Psychology; Social Work | Mental health professionals: psychologists; social workers (n = 79) | Four case vignettes designed to test multiple cognitive or diagnostic errors: Racial bias; base rate neglect; search satisficing; diagnosis momentum (anchoring); overconfidence bias. Characters’ ethnicity was also manipulated to be either Caucasian or African American across all the case vignettes. Participants were also randomly assigned to a debiasing intervention or a control group. Participants were asked to diagnose the case vignettes and provide a confidence rating. | Diagnosis; Treatment | Participant diagnostic accuracy for each of the four vignettes represented the presence or absence of the biases under consideration. Differences in the responses for vignettes depicting African American vs Caucasian characters was predicted to represent racial bias. Where diagnostic accuracy did not align with confidence ratings, this represented an overconfidence bias. | Racial bias; Base rate neglect; Search satisficing; Diagnosis momentum (anchoring); Overconfidence bias. | Y |
| Jenkins & Youngstrom, 2016; USA^70^ | Journal article | Psychology; Social Work | Mental health professionals: psychologists; social workers (n = 137) | Four case vignettes designed to test multiple cognitive or diagnostic errors: Racial bias; base rate neglect; search satisficing; diagnosis momentum (anchoring). Characters’ ethnicity was manipulated to be either Caucasian or African American across all the case vignettes. Participants were randomly assigned to a debiasing intervention or a control group. | Diagnosis; Treatment | Participant diagnostic accuracy or each of the four vignettes represented the presence or absence of the biases under consideration. Differences in the responses for vignettes depicting African American vs Caucasian characters was predicted to represent racial bias. | Racial bias; Base rate neglect; Search satisficing; Diagnosis momentum (anchoring) | Y |
| Joachim, 1981; USA^71^ | PhD Dissertation | Psychology | Clinical psychology graduate students (n = 54) | Four clinical case vignettes: two represented cases of mild psychopathology and two of severe psychopathology. The sex attributed to each character was systematically varied. Participants rated each case on 11 common criteria of mental health, combined to form a diagnostic scale. | Diagnosis | Predicted that: clinician sex would alter any diagnostic sex bias; case severity and case sex would interact to affect diagnostic ratings; female vignette characters would receive more severe ratings than male vignette characters for both mild and severe conditions. | Sex bias | Y |
| Johannesen, 2012; USA^72^ | Doctor of Psychology Thesis | Psychology | Graduate and professional psychologists who have worked with children for a minimum of two years (n = 27) | Child client drawings were presented to participants paired with either no history, an inaccurate history or an accurate history. Participants were asked to make diagnostic inferences about the drawing in each case. | Diagnosis | Predicted that client history, whether accurate or not, would influence the diagnostic inferences made about the client’s drawing, indicating confirmation bias. | Confirmation bias | Y |
| Jones, 1982; USA^73^ | Journal Article | Psychology | Clients (n = 164); Therapists (n = 136) | Clients who attended more than 30 therapist sessions were selected and grouped into four groups: black clients with white therapists; black clients with black therapists; white clients with white therapists; white clients with black therapists. Therapists were asked to complete several measures relating to patient descriptions and therapy outcomes. | Assessment; Prognosis | Predicted that black clients would be reported as having more negative assessment and therapy outcomes compared to white clients overall, and that assessments and therapy outcomes would be a function of racial similarity between the client and the therapist. | Racial bias | Y |
| Kolker, 1994; USA^74^ | PhD Dissertation | Psychology | Doctoral level psychologists (n = 372) | Each participant read one of four versions of a clinical case vignette depicting cases with differing symptomology that either met or did not meet the criteria for a diagnosis of Narcissistic, Histrionic or Antisocial Personality Disorders. Sex of the vignette character was altered across the cases (18 versions in total). Participants made a diagnosis and completed multiple questionnaires. | Diagnosis | Predicted that participants would display a diagnostic sex bias for Narcissistic Personality Disorder (male>females), which could not be explained by the gender base rate hypothesis (using base rates outlined within DSM-III-R). | Sex bias | Y |
| Koscherak & Masling, 1972; USA^75^ | Journal Article | Psychology | Graduate clinical psychologists (n = 41) | Participants rated fabricated Rorschach responses on personality scales and also wrote short assessment summaries. Half of the participants were told that the responses were made by a client of lower class status, and the other half were told the client was middle class | Diagnosis | Predicted that more negative diagnosis would be made for the client described as lower class. | Socioeconomic status bias | Y |
| Langer & Abelson, 1974; USA^76^ | Journal Article | Psychology | Behavioural and analytic/traditional therapists (n = 40) | Participants were shown a videotaped interview depicting a client and therapist. One half of the participants were told the client was a ‘job applicant’ and the other half were told he was a ‘patient’. Participants were asked to provide a brief, free-response description of the interviewee. | Assessment | Predicted that the client’s presenting issues would be rated as more severe when they were labelled as ‘patient’, compared to when they were labelled as ‘job applicant’. Also predicted that these differences would be more apparent for traditional versus behavioural therapists. | Labelling bias | Y |
| Lee & Richer, 1992; Canada^77^ | Journal Article | Psychology | Counsellors (n = 24) | Participants interviewed a normal, coached client for 30 minutes. Before the interview, one group received information indicating that the client had experienced depression in the past, and the other group received no such information. Immediately after the interview, participants rated the client on depressive symptoms. The occurrence of confirmatory statements and questions were obtained from coding an audiotape of the interview. | Assessment | It was predicted that counsellors who received pre-interview information that a client had previously experienced depression would, when compared with those who received no such information: rate the client more in accordance with prototypical descriptions of depression; use more confirmatory statements and questions concerning depression during the interview. | Inferential error; Confirmation bias | Y |
| Lee et al., 1995; Canada^78^ | Journal Article | Psychology | Graduate counselling psychologists (n = 52) | Participants viewed a videotaped interview. Before the interview, one group received information indicating that the client had experienced depression in the past, and the other group received no such information. Immediately after the interview and at two other time points, participants completed a recognition checklist intended to measure the degree to which participants correctly recognised information presented in the videotape. Participants also rated the client on depressive symptoms and provided an assessment of confidence in their own clinical impression. Participants were also assessed for their level of depression-related schema. | Assessment | Predicted that participants with well developed schema about depression and who receive a pre-interview suggestion about the videotaped client, inomparison with those with less developed schema and did not receive the suggestion, will: remember more confirmatory information; rate the clients behaviour more in the direction consistent with the pre-interview suggestion, and; have a higher confidence in their judgement. | Confirmation bias | Y |
| Levy & Kahn 1970; USA^79^ | Journal Article | Psychology | Graduate clinical psychology students and experienced psychologists (n = 130) | Participants rated one version of three Rorschach protocols, combined with case histories that were identical excepting in the socioeconomic status (lower, upper, none provided) of the depicted client. Participants were asked to complete a number of rating scales relating to assessment, diagnosis, prognosis and treatment for the depicted client. | Assessment; Diagnosis; Prognosis; Treatment | Predicted that Rorschach interpretation would elicit more  negative assessment, diagnoses, prognosis and treatment responses for patients with lower-class than for patients with middle-class social histories; and experienced interpreters would be less biased than inexperienced ones. | Socioeconomic status bias | Y |
| Lowry & Higgins, 1979; USA^80^ | Journal Article | Psychology; Social Work | Professional psychotherapists, including psychology, social work and psychiatry disciplines (n = 120) | Each participant rated three cases relating to: depression; schizophrenia; alcoholism. Cases depicted either a female or male character. Participants rated cases for severity of disturbance and made endorsements of six possible treatment options. | Assessment; Treatment | Predicted that severity of disturbance ratings and endorsement of treatment ratings would alter with the sex of the clients across the three cases. | Sex bias | Y |
| Luepnitz et al., 1982; USA^81^ | Journal Article | Psychology | Graduate psychology students (n = 40) | Participants were asked to view one of four videotapes depicting intake interviews with clients who met the criteria for alcohol abuse. The four versions reflected variations in race (black, white) and socioeconomic status (lower, upper). | Diagnosis | Predicted that diagnosis of alcohol abuse would be lower for white and upper socioeconomic clients. | Socioeconomic status bias | Y |
| Mack, 1998; USA^82^ | Journal Article | Psychology | Psychologists (n = 81) | Participants were asked to read one version of two clinical vignettes, one that met the criteria for antisocial personality disorder and one that did not. Each vignette was manipulated to depict a different client race (white, African American, Neutral). The race of the participants was also included as a variable. Participants were asked to assess the severity of presenting antisocial traits, psychopathy score, diagnosis of antisocial personality disorder (APD), prognosis, treatment recommendations, and perceptions of dangerousness. | Assessment; Diagnosis; Treatment; Prognosis | Predicted that the Black vignettes, as compared to the White and Neutral  vignettes, would be perceived as having higher severity level of pathology, given less adequate treatment recommendations, and receive higher ratings of dangerousness. In addition, the Caucasian psychologists were  expected to provide higher ratings of pathology than the African American psychologists. | Socioeconomic status bias | Y |
| Markowitz, 1989; USA^83^ | PhD Dissertation | Psychology | Clinical psychology graduate students (n = 42) | Participants viewed two half hour videotaped interviews. One interview was of a psychologist interviewing a mother and the other was an interview with a father. Of the same 2-year old. Both parents were depicted by trained actors. Participants either watched the interview with the mother first, or the father first. Participants rated each parent and were asked to make a custodial decision about whether the mother or father should take custody of the child. | Child placement | Predicted that the order that participants watched the two interviews would alter both the ratings and the custody decision. | Order effects | Y |
| Martin, 2001; USA^84^ | PhD Dissertation | Psychology | Psychologists with and without advanced clinical training (n = 80) | Participants were randomly assigned to  receive either specific instructions aimed at increasing disconfirmatory information  search strategies or received no such instructions. Participants then interviewed a mock therapy client. At three points across the session, participants provided a diagnostic hypothesis, rated their confidence in the accuracy of that hypothesis, and listed questions they wished to ask in order to clarify their hypothesis. | Assessment; Diagnosis | Predicted that more confirmatory questions would be asked by: non-trained vs trained participants; participants who did not receive the expanded, disconfirmatory instruction set vs those who did; participants who endorsed a ‘correct’ diagnosis and; participants who were more confident in their diagnostic hypothesis. | Confirmation bias | Y |
| Matthews, 1987; USA^85^ | Doctor of Social Work Thesis | Social Work | Social workers (n = 301) | Participants viewed eight family case vignettes that varied by the race (Jewish-American, Afro-American, Polish-American, Puerto Rican) and level of acculturation (high, low). Participants race and level of experience was included as a variable. Participants were asked to complete an assessment measure that included 20 indicators relating to diagnostic, assessment, intervention, service planning and other clinical issues. | Diagnosis; Assessment; Treatment | Predicted that client race and level of acculturation would be associated with differences in clinical judgement. These differences will also moderated by clinician experience, clinician race and type of client race. | Racial bias | Y |
| McAshen 2018; USA^86^ | PhD Dissertation | Psychology | Professional counsellors (n = 306) | Participants were randomly assigned to one of six vignettes featuring a young woman who engages in restrictive eating behaviours and over-exercising. Vignettes varied only by weight (Low, High) and ethnicity (White, Black, Hispanic). | Assessment; Treatment | It was predicted that participants would rate the vignette character’s symptomology as less severe when she is described as not significantly underweight, and also be less likely to recommend medical follow up. It was also predicted that symptomology ratings and recommendations for medical follow up would differ with the ethnicity of the vignette character. | Racial bias; Stereotype bias (weight) | Y |
| McCormick, 2000; USA^87^ | PhD Dissertation | Psychology | Psychologists (n = 207) | Participants were presented with base rate information for a stereotype-neutral, stereotype-consistent or stereotype-inconsistent disorder with information about a client. Each participant read one case vignette, and was asked to determine the probability that the character had the disorder, and provide a confidence rating for their response. Cases varied by sex (male, female, neutral), stereotype (Disorder x Sex), and base rate (Low 20%, Medium 50%, High 80%). | Diagnosis | Predicted that participants would: overestimate probability that the client has the condition for the low base rate condition and underestimate probability in the high base rate condition; judge females as more likely to have Histrionic Personality Disorder; judge males more likely to have Antisocial Personality Disorder; judge females as more likely to have personality disorders in general (beyond what can be explained by the utilisation of base rates).. | Gender bias (sex bias); Base rate fallacy | Y |
| Meitus et al., 1973; USA^88^ | Journal Article | Speech Pathology; Audiology | Audiology and speech science graduate student clinicians (n = 30) | Videotaped recordings of ‘talkers’ displaying some disorder of speech articulation, including problems attributable to neurologic factors, structural anomalies and functional causes were evaluated by 30 clinicians (participants). 10 were exposed *a priori* to a positively biased case history, 10 to a negatively biased case history, and 10 saw no case history prior to evaluation. Participants evaluated the talkers via a formal phonetic inventory instrument and on a five-point articulatory-proficiency scale. A further series of four-point scaes were undertaken to determine prognostic judgements and treatment plans. | Assessment; Prognosis; Treatment | Predicted that the case history bias would influence participants’ judgments of the severity of the speech disorder, and the formulation of prognostic hypotheses and therapeutic programmes. | Examiner bias | N |
| Merluzzi & Merluzzi, 1978; USA^89^ | Journal Article | Psychology | Graduate counselling students (n = 86) | Participants read four (plus four dummies) intake case summaries. For one group of participants, the clients in those four summaries were labelled black, for a second group they were labelled white, and for a third group there were no racial labels. Participants rated the cases on 11 positive to negative dimensions related to client characteristics, counselling readiness, environmental effects on the client and predicted outcome. Participants were also assessed for clinical experience, their own personal contact with minorities, and their physical social distance to minorities. | Assessment; Prognosis | Predicted that race of the intake case summary would be associated with differences in clinical assessment. Also predicted that participant variables may influence these outcomes. | Racial bias | Y |
| Messier, 1997; USA^90^ | PhD Dissertation | Psychology | Practicing psychologists (n = 203) | Participants were randomly assigned a single vignette in which the case character depicted varied in age (35,75 years) and health status (good, chronically ill, terminally ill). Participants’ were asked to diagnosis, assess the risk of suicide, provide a prognosis for the illness and make treatment recommendations for the vignette character. They were also asked to complete questions relating to their own understanding about old age and disability. | Diagnosis; Prognosis; Treatment | Predicted that participants’ diagnosis, assessment of risk of suicide, prognosis of the illness and treatment recommendations for the vignette character, would differ with age and health status. It was also predicted that a participant’s understanding about old age and disability may influence these outcomes. | Age bias; Health status bias | Y |
| Michaud, 1994; USA^91^ | PhD Dissertation | Psychology | Psychologists (n = 260) | Participants were randomly assigned to two groups. The groups were allocated case vignettes that varied in the prior diagnostic information provided about each case. Two additional vignettes varied only in the gender and racial identity of the case character. Participants were asked to provide a diagnosis and reason for the diagnosis. | Diagnosis | It was predicted that the groups would differ in the diagnosis they provided, depending on the prior diagnostic information they received with the case vignette. It was also predicted that females (not males) and African American (not Caucasian) cases would receive more severe diagnoses. | Gender bias (sex bias); Racial bias; Confirmation bias | Y |
| Mohr et al., 2009; USA^92^ | Journal Article | Psychology | Psychotherapists (n = 108) | Participants read one version of a fictitious client intake report that varied only in that the male client was depicted as either heterosexual, gay or bisexual. Participants rated the case for the salience of a variety of clinical issues (some of which were related to bisexual stereotypes), global psychological functioning, and anticipated reactions to the client. | Assessment | Predicted that participants in the bisexual condition would give high relevance ratings to clinical issues that were related to bisexual stereotypes, but not to clinical impressions unrelated to bisexual stereotypes. Predicted that this outcome would be mediated by participant beliefs and anticipated reactions to the character in the vignette. | Sexual orientation bias | Y |
| Mosier, 2014; USA^93^ | PhD Dissertation | Psychology | Graduate clinical and counselling psychology student (n = 204) | Participants received one of five diagnostic vignettes, each describing individuals displaying identical symptoms of both Borderline and Antisocial Personality Disorder, but altered by gender and sexual orientation. Participants were asked to provide a diagnostic impression from categorical or dimensional trait perspective, and undertook measures of attitudes towards women and homosexual individuals. | Diagnosis | Predicted that men would be more likely to be diagnosed with Antisocial Personality Disorder and women with Borderline Personality Disorder. It was also predicted that sexual orientation stereotypes would influence diagnostic impression, e.g. individuals seen as displaying more traditionally feminine attributes (heterosexual women and gay men), would receive more frequent diagnoses of Borderline Personality Disorder. | Gender bias (sex bias); Sexual orientation bias | Y |
| Mumma, 2002; USA^94^ | Journal article | Psychology | Study 1: Doctoral level clinical psychologists (n = 73). Study 2: Doctoral level clinical psychologists (n = 46) | Study 1: Participants were randomised to one version of two videotaped client interviews. Information provided within the interviews was manipulated using a 2x2x2 factorial design to test for anchoring effect, expectancy effect and halo effect. Participants recorded symptom severity ratings for Major Depressive Disorder after watching the relevant diagnostic portion of the interview. Study 2: Participants were randomised to one version of two videotaped client interviews. Information provided within the interviews was manipulated to test for the anchoring, expectancy and halo effects. Participants recorded symptom severity ratings for Major Depressive Disorder after watching the whole interview. | Assessment | Study 1: Prior and current diagnostic criteria was presented at clear-cut or near-threshold levels to assess the anchoring effect, expectancy effect and halo effect, and this information was predicted to alter severity ratings provided by participants. Study 2: Level of depressive non-verbal behaviour and essential diagnostic information was presented at clear cut or threshold levels, and information about prior depression was presented as either subthreshold or absent. These manipulations were expected to influence symptom severity ratings. | Anchoring effect; Expectancy effect; Halo effect | Y |
| Nalven et al., 1969; USA^95^ | Journal Article | Psychology | Clinical and school psychologists (n = 320) | Participants responded to one variation of a questionnaire outlining Wechsler Intelligence Scale for Children (WISC) scores. Background information was varied by age (8/14 years), sex (male/female), race (Caucasian/Negro) and socioeconomic status (lower/middle). Participants were asked to score the ‘true IQ’ of the test results. | Assessment | Predicted that variation in participants’ ‘true IQ’ scores would be due to differences in the background information (age, sex, race, SES) | Age bias; Socioeconomic status bias; Racial bias; Sex bias | Y |
| New, 2005; USA^96^ | PhD Dissertation | Psychology | Psychologists (n = 2) | Investigated whether halo effect would skew  highly trained clinicians' scoring of the Marschak Interaction Method (MIM) using the Marschak Interaction Method Rating System (MIMRS). Before rating pre- and post- treatment client videotapes, one rater was given incorrect information about the videotape being pre- or post- treatment. The other rater did not receive the biased information. | Assessment | Predicted that the rater who received biased information about treatment status would have significantly different rating scores than the rater who did not receive biased information. | Halo effect | N |
| O’Reilly, 1986; USA^97^ | PhD Dissertation | Psychology | School psychologists (n = 40) | Participants evaluated a report describing a child who is being referred for either a Gifted or Learning Difficulties placement consideration. All assessment data were identical, and participants were asked to classify the child as either being Gifted or having a Learning Disability. | Assessment | Assessments of either Gift or Learning Difficulty made by participants were expected to be consistent with the information provided within the referral report. | Confirmation bias | Y |
| Oyster-Nelson & Cohen, 1981; USA^98^ | Journal Article | Psychology | Psychologists (n = 119) | Participants evaluated one of three clinical vignettes that portrayed either a male-appropriate, female-appropriate, or neutral problem. The sex (male, female) of the client was manipulated for each problem. Participants were asked to complete a questionnaire relating to the severity of the client’s presenting problem and treatment choices. | Assessment; Treatment | If sex role stereotyping occurred, it was predicted that for the male client, the relationship problem (female-appropriate) should be seen  as more serious than the achievement-related problem (male-appropriate), and for the female client, the opposite pattern would be found. In addition, clinical bias, if existing, would be  demonstrated by the psychologists' differential recommendation of specific treatment modalities as a function of the client's sex. | Sex bias; Sex role stereotype bias | Y |
| Padol & Salvia, 1976; USA^99^ | Journal Article | Speech Pathology | Graduate speech pathology students (n = 60) | Participants were randomly assigned to one of four treatment groups. All read identical case studies about a child born with a cleft. One of two photographs was attached to each case study. One was an accurate photograph of a girl with post-operative  disfigurement. The second was of the same girl, but it had been retouched to eliminate the disfigurement. Half of the participants in each photograph condition listened to a recording of a normal speaker, and half listened to a nasal speaker. Participants then assessed the speech of the child and made a treatment recommendation. | Assessment; Treatment | Predicted that the appearance of a facial disfigurement in the attached photo would influence the speech assessment and recommendations for treatment. | Expectancy effect relating to a facial disfigurement | Y |
| Parmley, 2006; USA^100^ | PhD Dissertation | Psychology | Psychologists (n = 102) | Participants received two case vignettes, followed by additional information about the case a week later. The additional information was either consistent or inconsistent with the previously presented diagnosis. Participants were asked to make a diagnosis after reading each vignette. Half of the participants also received information about the confirmation bias before receiving the additional information. | Diagnosis | Predicted that participants would be more likely to retain their original diagnosis, regardless of whether another diagnosis was more appropriate. | Confirmation bias | Y |
| Paster, 2012; USA^101^ | Doctor of Psychology Thesis | Psychology; Social Work | Mental health practitioners (n = 62) | Participants assessed three short case vignettes providing background information for a client and an ambiguous clinical presentation for a DSM-IV diagnosis. Participants made a diagnosis and were asked a series of questions relating to the diagnosis, including a weighting of symptoms that informed the final diagnosis. | Diagnosis; Assessment | Predicted that the race of the vignette character would produce a bias in participants’ diagnoses with vignettes depicting Black or Latino characters more likely to receive certain diagnoses, despite the ambiguous case description. Also predicted that the weightings for particular symptomology would differ between vignettes depicting Black/Latino vs White characters. | Racial bias | Y |
| Patterson, 1982; USA^102^ | PhD Dissertation | Psychology | Professional psychologists; graduate clinical psychology students (n = 64) | Participants were administered two diagnostic vignettes that were designed to look like one diagnosis initially and end up looking like another. Half the subjects were randomly assigned to a condition where they were asked to generate hypotheses during pauses in symptom presentation. The second group was not asked to do this. Participants made a diagnosis of the case vignette. | Diagnosis | Predicted that participants would display a primacy effect towards the initial diagnostic information, such that the diagnosis more often conformed to the information provided earlier in the case vignette. It was also predicted that the primacy effect would not be shown in the hypothesis generation group. | Primacy effect | Y |
| Perlick & Atkins, 1984; USA^103^ | Journal Article | Psychology | Clinical psychologists (n = 36) | Participants listened to a tape recording of a standard psychiatric interview conducted with an actual patient. Before listening to the interview, participants were told the patient was 55 or 75 years old, or asked to provide a diagnosis based on symptomology alone (no age condition). Participants were asked to make a diagnosis of the patient using a questionnaire, and were also asked to rate their confidence in the diagnosis. | Diagnosis | Predicted that participants would diagnose more organic (reflective of senile dementia) and less depressive pathology (mental health) when the patient’s age was reported as 75 than when it was reported as 55 or not reported. | Age bias | Y |
| Peyser, 1984; USA^104^ | PhD Dissertation | Psychology | Practicing psychologists; graduate psychology students (n = 64) | Identical responses to the Comprehension subtest of the Wechsler Intelligence Scale for ChiIdren-Revised were audiotaped on  separate cassettes by two children, one with and one with no speech impairment. Participants were randomly assigned to score the responses on one of the tapes. Sixteen of each group of participants scored the responses by the child with the speech impairment; sixteen of each group scored the responses of the child with no speech  impairment. | Assessment | Predicted that the mean rating, as well as the range of ratings across participants, for the child with and the child without a speech impairment would differ. | Examiner bias; Expectancy effect | Y |
| Pfeiffer et al., 2000; USA^105^ | Journal Article | Psychology | Graduate psychology students (n = 72) | Participants read a fictional written referral from a physician which outline one of three problems relating to a hypothesis condition: therapists’ own hypothesis; high-plausibility hypothesis; low plausibility hypothesis. Participants were further assigned to a low and high accountability conditions where they were told that their decision would be reviewed by a supervisor, or not. Participants then watched a videotaped interview between an actor and trained social worker. Participants were asked to supply an issue to discuss in therapy in the form of a diagnosis, provide a list of verbal and non-verbal cues, provide therapy questions and reasoning behind then, and complete a post-interview questionnaire. Participant responses were rated as consistent, neutral or inconsistent with the target diagnosis. | Diagnosis | The study predicted that the confirmation bias would not occur as a result of referral information. Predicted that participants would attend to, gather, and interpret information in a more consistent manner when testing their own hypotheses and highly plausible provided hypotheses than when testing less plausible provided hypotheses. It was also proposed that therapists would be less consistent in their decision making when being held highly accountable for their decisions than when being held minimally accountable. Third, it was hypothesized that when therapists were provided with less plausible hypotheses, they would develop their own hypotheses that were more compatible with the client's presentation. | Confirmation bias | Y |
| Pickholtz, 1977; USA^106^ | PhD Dissertation | Psychology | School psychologists (n = 96) | Participants read individual fictitious psychological reports of borderline intelligent children who differed only in terms of the assigned racial-ethnic label and mean achievement grade scores. Participants rated the children in terms of assigning the children to four separate types of educational classifications (EMR, Learning Disability, ED, and NORMAL), and the degree of placement integration which the children  warranted. | Assessment; School placement | Predicted that a child’s racial-ethnic and achievement grade scores would influence participants’ classification label and child education placement recommendations. | Racial bias | Y |
| Prout & Frederickson, 1991; USA^107^ | Journal Article | Psychology | School psychologists (n = 97) | Two case vignettes presented two separate problems (internalising externalising), and were manipulated for sex (female, male) of the client. Participants read one case vignette and were asked to answer a series of questions relating to the extent of the perceived disturbance and the importance of intervention. | Assessment; Treatment | Predicted that participants’ rating for perceived disturbance and importance of intervention would alter with sex of the portrayed case character. | Sex bias | Y |
| Ramig, 1982; USA^108^ | Journal Article | Speech Pathology | Undergraduate and graduate speech pathologists (n = 64) | Participants were divided into four groups: group I evaluated only an audiotrack; group II evaluated an audiotrack and were told speakers have cleft palate; group III received an audio and visual track were told they were rating cleft palate speakers; group IV evaluated audio and visual track and were told they were rating cleft palate speakers who had been described as ‘hypernasal’. Audio and visual tracks depicted ten male speakers with cleft lip and/or palate. | Assessment | Predicted that a ‘cleft-palate’ label, any facial disfigurement associated with the cleft lip and/or palate, and the description ‘hypernasal’ would affect speech ratings. | Labelling bias; Expectancy effect | Y |
| Ray et al., 1987; USA^109^ | Journal Article | Psychology | Clinical psychologists (n = 192) | Each participant assessed four separate vignettes describing four different psychological disorders. The vignettes characters were manipulated such that their ages differed across the vignettes. Participants were asked to respond to questions relating to how ideal the clients were for psychologist’s practice, the clients’ prognoses, and were also asked to recommend a treatment plan. Participant age was also recorded. | Assessment; Treatment; Prognosis | Predicted that older clients would be evaluated as less desirable, receive poorer prognoses, and be chosen for psychotherapy less often. Also predicted that younger psychologists would view younger clients as more ideal to work with, compared to their older counterparts. | Age bias | Y |
| Riensel, 1976; USA^110^ | PhD Dissertation | Social Work | Social workers (n = 207) | Participants read one of four versions of two separate experimental case analogues where socioeconomic status (lower class/upper class) and race (white/black) of the depicted client was systematically varied. Participants then responded to an inventory of judgement items relating to assessment and treatment. | Assessment; Treatment | Predicted that participant judgements would be significantly different for lower class clients than for upper class clients, and would also differ by race of the depicted client. | Racial bias; Socioeconomic bias | Y |
| Roades, 1994; USA^111^ | PhD Dissertation | Psychology | Clinical psychologists (n = 524) | Participants were asked to provide diagnostic and clinical ratings for one of two case histories; depressed or alcohol. For each case, one of five client types were depicted: African American female; White female; African American male; White male, and; no information about race or gender. | Diagnosis | It was predicted that client gender and race would affect clinical diagnosis for both the alcohol and depressed case vignettes. | Racial bias; Gender bias (Sex bias) | Y |
| Robertson & Fitzgerald, 1990; USA^112^ | Journal Article | Psychology | Practicing counsellors and therapists (n = 47) | Participants were randomly assigned to view one of two versions of a videotaped simulation of a depressed, white male client. The tapes were identical excepting that in one version the male was portrayed as gender traditional and in the other version was gender non-traditional. Participants were asked to respond to the client verbally during the tape, evaluate the client on multiple dimension after watching the tape, assign a diagnosis and outline a possible treatment plan. | Assessment; Diagnosis; Treatment | Predicted that the gender role assigned to the character would influence the participants’ verbal responses, evaluations, diagnoses and treatment plans. Specifically, it was predicted that for the non-traditional male, counsellors would be more directive in their approach, rate their problems as more severe (indexed as diagnosis), ascribe more situational factors as a possible cause of the issues (e.g. domestic responsibilities), and view these clients as more feminine. | Gender (Sex) role stereotype bias | Y |
| Rotem-Lehrer, 2016; Israel^113^ | Journal Article | Physical Therapy | Physical therapists (n = 120) | Participants received a single short description of the clinical background of a volunteer. One of three different sets of information were provided participants: a true description of the case, and two fictional descriptions designed to induce the anchoring bias (moderate, substantial). Participants were asked to provide a Wrist range-of-motion measurement. | Assessment | Predicted that wrist range-of-motion measurements would be affected by the information provided within fictitious clinical background information. | Anchoring | Y |
| Routh & King, 1972; USA^114^ | Journal Article | Psychology | Clinical psychologists (n = 15); Undergraduate psychology students (n = 32) | Participants were asked to read 24 separate paragraphs about different clients. These were randomised across participants by occupation (either lower or middle class), behavioural descriptions (normal, neurotic, or psychotic), and mood adjectives (neutral or depressed) with the constraint that each of the 12 combinations of the 2 X 3 X 2 design was represented by two paragraphs. Participants were asked to provide a numerical response indicated how much they believed the client in the description was in need of professional help. | Assessment | Predicted that participants’ responses would change with socioeconomic status, and that this would also be mediated by the behavioural descriptions, mood adjectives and professional experience of the participants. | Socioeconomic status bias | Y |
| Sattler et al., 1970; USA^115^ | Journal Article | Psychology | Psychology graduate students (Study 1, n = 15; Study 2, n = 8) | Study 1: Participants were asked to score written responses to the Wechsler Intelligence Scale for Children (WISC) and Wechsler Adult Intelligence Scale (WAIS). Three intellectual levels were used: a level indicating that examinees were bright; a level indicating that examinees were dull, and an unspecified level in which no information was provided about the examinees' ability level. Study 2: Participants scored responses to the WISC/WAIS after watching a tape recording depicting ambiguous, average and superior responses, in different orders, for each of the four treatment groups. | Assessment | Predicted that participants would give more credit to responses produced by the examinees said to be bright than to the same responses produced  by examinees said to be dull, and examinees who have produced other "bright" responses than to examinees who have produced other "dull"  responses. | Halo effect | Y |
| Saxon & Spitznagel, 1992; USA^116^ | Journal Article | Psychology | Vocational rehabilitation counsellors (n = 64) | Participants were asked to assess four vocational evaluation reports. The ages of the clients depicted within the four reports varied across the participants. Client age represented one of four age continua: 30-39; 40-49; 50-59; 60-69 years of age. Participants were asked to provide a rating of the feasibility of the client for rehabilitation services. | Assessment | Predicted that client age would alter the feasibility rating for rehabilitation services. | Age bias | N |
| Seem & Johnson, 1998; USA^117^ | Journal Article | Psychology | Graduate counselling students (n = 210) | Participants read one of four versions of two case vignettes. One vignette described a female gender role description and the other a male gender role description. Each of these was varied by sex (female, male). Participants were asked to answer four free-response measures in relation to information gathering, formulation of the problem, and treatment goals. | Assessment; Treatment | It was predicted that when sex and gender roles were not congruent, this would produce bias, such that clinical assessment outcomes would be more negative for these vignettes. | Gender (Sex) role stereotype bias | Y |
| Shenkel, 1979; USA^118^ | Journal article | Social Work | Social welfare students (n = 63) | Participants were randomly assigned to one of nine conditions involving listening to a taped therapy session. Participants in the ‘before-tape’ condition received a diagnosis in which a previous clinician stated that they were very confident or tentative about the diagnosis, and the client’s issues were stated as either situational or personality based. Participants assigned to the ‘after-tape’ condition listened to the tape and then received one of the two previous clinician’s diagnoses. Participants were each asked to write a subjective evaluation of the client and complete an objective scale measurement. | Assessment | It was predicted that participants’ subjective and objective assessments of the client taped therapy session would change depending on: the situational/personality diagnosis of the previous clinician (fundamental attribution error); receiving the previous diagnostic report before or after listening to the taped therapy session (recency effect); and the confidence expressed by the previous clinician. | Fundamental attribution error; Recency effect | Y |
| Sieracki, 2010; USA^119^ | PhD Dissertation | Social Work | Social workers (n = 231) | The study used a single vignette, portraying a youth and foster family scenario, with three experimentally manipulated variables (Caucasian/African American, high/low socioeconomic status, wraparound services/treatment as usual), resulting in eight randomly assigned conditions. Participants were randomly assigned to a condition and read one version of the vignette. Participants were asked to make a recommendation for community or residential care, complete a youth assessment measure, and service recommendations. | Assessment; Child placement | It was predicted that the race, socioeconomic background, and type of service provision would influence ratings for youth psychopathology and risk behaviours, recommendations for residential or community placement, and recommendations for additional services. It was also predicted that professional experience may moderate the outcomes. | Socioeconomic status bias; Racial bias | N |
| Smith, 2013; USA^120^ | PhD Dissertation | Psychology | Clinical psychology doctoral students; licenced psychologists (n = 90) | Participants read the same vignette and were randomly assigned to one of four conditions: black race/no salience instructions; black race/salience instructions; white race/no salience instructions; white race/salience instructions. Vignettes depicted either a black or white character, and participants either received or did not receive information about racial bias. Participants were asked to provide a rating of the future likelihood of violence. | Assessment | The primary prediction was that violence ratings would differ across groups, with vignettes depicting black characters being associated with higher violence scores. | Racial bias | N |
| Snyder 1977; USA^121^ | Journal Article | Psychology | Psychodynamic and behavioural psychologists (n = 40) | Participants observed a taped interview. Half of the participants were told that the interviewee was a job applicant and the other half were told the interviewee was a patient. Participants were asked to respond to questions in order to ascertain whether they assessed the client’s problems stemmed from person-based or situational factors. | Assessment | Predicted that participants who were told the client was a patient, would rate the client’s problem as more person-based, rather than situational. It was also predicted that the bias would be less pronounced for behavioural vs psychodynamic trained participants. | Labelling bias | Y |
| Spaanjaars et al., 2015; Netherlands^122^ | Journal Article | Psychology | Psychologists (n = 126) | Moderate and very experience clinicians were randomly assigned to reading a referral letter suggesting either depression or anxiety. They then read psychiatric report about a depressed patient, and gave a preliminary and final diagnosis. Results | Diagnosis | Predicted that diagnoses suggested in referral letters, influence judgments made by clinical psychologists with different levels of experience. | Anchoring effect | Y |
| Spengler et al., 1990; USA^123^ | Journal article | Psychology | Counselling psychologists (n = 165) | Participants assessed a vignette that described a vocational problem, either alone or in conjunction with a personal problem of a half, equal or double the severity of the vocational problem. Participants were asked to complete a range of measures assessing their professional preferences for career and personal problems, and answer a range of questions relating to assessment, diagnosis and treatment of the client. | Diagnosis; Assessment; Treatment | Predicted that assessment, diagnosis and treatment judgements of participants would differ, such that overshadowing of vocational issues would be positively associated with the severity of the co-occurring personal issues. It was also predicted that counsellor preference for personal problems would be associated with a greater likelihood of the vocational overshadowing bias. | Vocational overshadowing bias | Y |
| Spengler, 2000; USA^124^ | Journal article | Psychology | Clinical and counselling psychologists (n = 146) | Participants read one of five case vignettes describing a client non-career and career-based problems. Non-career problems were manipulated and ranged from non-severe to severe. Participants were asked to complete a range of measures assessing their professional preferences from career and personal problems and cognitive complexity about psychotherapy versus career counselling, and answer a range of questions that formed an aggregate vocational clinical judgement score for the client. | Assessment | Predicted that vocational overshadowing bias would occur for the vignettes with increasing severity of the non-career problems, such that the vignette characters’ career concerns would be underemphasised when the severity of the co-existing non-career problem was equal to or less than the career problem. It was also predicted that counsellor preference and training would be associated with a greater likelihood of the vocational overshadowing bias. | Vocational overshadowing bias | N |
| Staal, 1993; USA^125^ | Doctor of Psychology Thesis | Psychology | Graduate clinical psychology students, practicing psychologists (n = 158) | Participants were asked to read one version of two transcripts depicting hypothetical client in an interview with a counsellor. Each transcript was varied by: order of presentation of salient material (early vs. late); sex (male vs. female). Level of education (graduate students vs. practitioner) and type of client (dangerous vs. non-dangerous) was also assessed. Participants were asked to rate the client and also assess their own self-perceptions and self-efficacy. | Assessment | Predicted that clinical rating would differ in relation to the order that salient information was presented, and with the sex of the client. Also predicted that the level of participant experience and type of client depicted would also alter clinical ratings. | Anchoring effect | N |
| Stevens, 1981; USA^126^ | Journal Article | Psychology | School psychologists (n = 24); Elementary school teachers (n = 27); Parents (n = 24) | All participants watched the same 3-minute film depicting six children in a school setting. Child behaviour was pre-assessed as being comparable for all six children. Six separate biographies were developed, such that the socioeconomic status of the child was indicated using the parental occupation and family dwelling. Hyperkinetic behaviour was assessed using a nine-point Likert-type rating scale for each of the target. | Assessment | Predicted that racial background and socioeconomic status would influence the participants’ ratings of hyperkinetic behaviour. | Racial bias; Socioeconomic status bias | Y |
| Strickland et al., 1988; USA^127^ | Journal Article | Psychology | Graduate clinical psychology students (n = 20) | Participants (black and white) were asked to view and rate six simulated clinical interviews of clients in which race and level of psychopathy (normal, neurotic psychotic) was systematically varied. Presentation of the vignettes was counterbalanced and randomised (e.g. five black and five white participants were shown a black paranoid schizophrenic client and a white manic disorder client). Each participant viewed and rated two normal (one black and one white), two neurotic (one black and  one white), and two psychotic (one black and one white) clients. Participants were asked to rate vignettes on six scales relating to client verbal skills, type and degree of psychopathology, appropriateness of therapy, likelihood of successful treatment and, socioeconomic status of the client. | Diagnosis | Predicted that black and white participants with comparable training and experience would rate black and white clients differently in terms of degree of psychopathology, level of verbal skills, appropriateness for therapy, likelihood of treatment success, and level of socioeconomic status. These differences were expected to be more pronounced when judging black clients. It was also predicted that black clients would be rated by white therapists as more disturbed, less verbally facile, less appropriate for therapy, less likely to benefit from treatment, and of a lower socioeconomic status. Black therapists, on the other hand, should rate black clients more positively. In addition, it was predicted that black and white therapists should not differ in their judgments on white clients. | Racial bias | Y |
| Swaggerty-Valdes, 2009; USA^128^ | PhD Dissertation | Psychology | Psychologists (n = 557) | Participants received one of nine variations of a clinical vignette: a control with no information provided about race or socioeconomic status (SES); African-American with no SES; white with no SES; African-American low SES; White with low SES; African-American with high SES; White with high SES; low SES with no race information; high SES with no race information. Participants were asked to provide a diagnosis, a global assessment of functioning, a symptom severity rating and a prognosis. | Diagnosis; Assessment; Prognosis | It was predicted that when the case vignette character was identified a as African American or from a low SES status, then participants would diagnose schizophrenia more than for the other conditions, and also differentially influence symptom severity ratings, assessments of global functioning and prognosis | Racial bias; Socioeconomic status bias | N |
| Tasby, 2008; USA^129^ | PhD Dissertation | Psychology | School psychologists (n = 308) | Participants read one of six variations of a vignette describing presenting problems for a single child consistent with a diagnosis of autism. The vignettes varied in the ethnicity presented (African-American, Caucasian, Hispanic) and socioeconomic status (low, high). Ethnicity was presented using a fictitious photograph. Participants were asked to complete a set of survey questions relating to the seriousness and unusualness of the presenting behaviours, the need to intervene, and provide a likely diagnosis. | Assessment; Diagnosis | It was predicted that participants’ perception of the seriousness and unusualness of the case characters’ behaviour, need to intervene, and their diagnosis would differ with the socioeconomic and ethnicity conditions. | Racial bias; Socioeconomic status bias | N |
| Teitler, 1995; France^130^ | Journal Article | Speech Pathology | Speech pathologists; otolaryngologists (n = 19) | Participants (raters) rated 30 subjects’ vocal folds according to a standardised evaluation form. Two case histories were constructed for each subject, one containing positive information about the patient and the other containing negative information. Raters were randomly divided into three groups. Rater groups I and II each contained the same amount of good and bad histories, randomly assigned to the subjects' vocal fold images. Rater group III did not receive case histories. | Assessment | Predicted that the assessment of the vocal folds on multiple vocal parametres would be more severe when subjects were presented with a bad case history, than for subjects presented with a good case history and when no case history was provided. | Examiner bias | Y |
| Teri, 1982; USA^131^ | Journal Article | Psychology | Psychologists (n = 237) | Participant sex was recorded and participants were also assessed using a measure of sex role styles (androgynous, undifferentiated, high masculine, high feminine). Participants read two case vignettes, one describing stereotypically male behaviour and the other stereotypically female behaviour. Sex (female, male) of the vignettes characters was systematically manipulated between the vignettes. Participants were asked to assess: maladjustment; prognosis; current  and expected functioning as a parent, as a spouse, socially, vocationally in a  high-pressure job, and vocationally in a low-pressure job. | Assessment; Prognosis | Predicted that client sex, participant sex, client sex role style and participant sex role style would produce differences in clinical judgements, including | Sex bias; Sex role stereotype bias | Y |
| Trachtman, 1968; USA^132^ | PhD Dissertation | Psychology | Male psychologists (n = 60) | Participants undertook measures so that they could be grouped into authoritarian level (high and low) and anxiety level (status threat, status reinforcement) such that four experimental groups were created. Participants were presented with four pairs of Rorschach reports that varied by socioeconomic status of the client outlined within a case history. Participants were asked to evaluate the records on functioning, diagnosis and prognosis. | Assessment; Diagnosis; Prognosis | Predicted that high authoritarian and high-status threat participants would be more likely to exhibit bias. Specifically, participants would assess greater pathology and less favourable prognoses to protocols depicted as lower class. | Socioeconomic status bias | Y |
| Umbenhauer, 1975; USA^133^ | PhD Dissertation | Psychology; Social Work | Mental health professionals, including psychiatrists, psychiatric residents, psychologists, psychology graduate students and social workers (n = | Each participant received one of four possible versions of each of two types of cases. The versions varied by race (white, black) and social class (upper or lower) so that the four conditions could be labelled as white-upper class, black-upper class, white-lower class, and black-lower class for each type of pathology (withdrawn or aggressive). Participants were asked to respond to questions relating to patient pathology, their feelings about the patient, and provide a ranking for the most appropriate treatment for the patient. | Assessment; Treatment | Predicted that clinical judgements would be less favourable for low socioeconomic patients and for black patients. Also predicted that profession type, experience and a range of other measures would moderate these outcomes. | Socioeconomic status bias; Racial bias | Y |
| Vail et al., 1970; USA^134^ | Journal Article | Social Work | Social casework students and practitioners (n = 170) | Participants read one version of an intake case description, where the case characters depicted varied by race black, white) and socioeconomic status (lower, upper). Participants completed a form to record clinical impressions to inform a psychosocial diagnosis, and responded to questions aimed at determining participants view of treatment potential and recommended level of treatment. | Assessment; Diagnosis; Treatment; Prognosis | Predicted that both race and socioeconomic status would be reflected in differences in clinical impressions, treatment potential and level of treatment. The study also expected that level of experience would mediate these effects. | Socioeconomic status bias; Racial bias | Y |
| Ventre, 1987; USA^135^ | PhD Dissertation | Psychology | Psychologists; psychology graduate students (n = 80) | Participants were randomly assigned to receive psychoeducational test results in one of two orders. Participants were asked to complete two rating scales to measure diagnostic impression. | Diagnosis | Predicted that participants would: differentially rate the extent to which clients were experiencing difficulty (diagnostic impression) as a function of the order in which psychoeducational test results were received; endorse the problem category consistent with their primacy information more often than other problems categories; rank tests corresponding to their primacy information as more important to diagnostic decisions, and; high-experience participants would be more susceptible to primacy effects. | Primacy effect | N |
| Waddington, 2000; UK^136^ | Journal article | Psychology | Clinical and trainee psychologists (n = 64) | Participants read a fictional referral letter describing a client either with or without adult attachment difficulties, and post-traumatic stress disorder. Participants were asked to recall information from the letter and also asked to provide information about their psychological orientation, training experience and confidence, and order of information in the letter. | Assessment | It was predicted that when referral letters contained attachment information, and when participants reported having a stronger attachment orientation, they would be more likely to recall attachment related information from the referral letter. | Availability bias | N |
| Walker & Spengler, 1995; USA^137^ | Journal article | Psychology | Practicing clinical and counselling psychologists (n = 450) | Participants completed a questionnaire that included a clinical vignette describing a man with major depression and a medical condition corresponding to one of three randomised experimental conditions: AIDS; Cancer; no medical condition. Participants were asked to rate the likelihood of 10 potential diagnoses, and 10 potential treatment options. Participants also completed an Attitude Towards AIDS victims scale. | Diagnosis; Treatment | Predicted that diagnostic and treatment ratings would differ between groups where the vignette character was also diagnosed with AIDS or cancer, and the no medical condition group. It was also predicted that participants’ attitudes to AIDS would alter their diagnostic and treatment ratings. | Diagnostic overshadowing | Y |
| Walling 1987; USA^138^ | PhD Dissertation | Psychology | Psychologists and undergraduate psychology students (n = 273) | Participants read one version of a case vignette and were asked to evaluate the client’s level of pathology and provide a prognosis. The case differed only in that the client was assigned one of two diagnostic labels (Adjustment disorder with depressed mood or Unipolar depression), and the timing of the presentation of these labels was varied. | Assessment; Prognosis | Predicted that the level of pathology ratings and prognosis would differ with the different labels and the timing of presentation. Also expected that participant experience would influence the level of pathology ratings and prognosis. | Labelling bias; Anchoring bias | Y |
| Warner, 1978; USA^139^ | Journal Article | Psychology | Psychiatrists, psychologists (n = 175) | Participants read one of two versions of a case vignette that described features of histrionic and antisocial personality. The case character was depicted as either male or female. Participants were asked to choose a likely diagnosis from a list, or make to make their own diagnosis. | Diagnosis | Predicted that when the case vignette depicted a male, an antisocial personality disorder diagnosis would be made more often, and when a female was depicted, histrionic personality disorder would be diagnosed more often. | Sex bias | Y |
| Warner, 1979; USA^140^ | Journal Article | Psychology; Social Work | Mental health professionals, including, psychiatrists, psychologists, psychiatric nurses, social workers, mental health workers, community counsellors, activity therapists (n = 173) | Participants read four clinical vignettes and were asked to choose from a list of potential diagnoses for each of the profiles. Each of the vignettes described one of four race-gender categories: patients in case A and C varied as white man, white woman, Chicano man or Chicano woman; patients in case B and D were white man, white woman, black man, black woman. | Diagnosis | Expected that both race and sex of the depicted character would predict differences in the diagnoses chosen across the four vignettes. | Sex bias; Racial bias | Y |
| Wendt & Tyson, 2018; Australia^141^ | Journal Article | Psychology | Psychologists (n = 121) | Participants viewed a case history depicting a depressed client in separate blocks of information. Participants either received or did not receive an introductory statement containing a diagnosis (anxiety or depression). Participants were asked to provide a diagnostic formulation. Participant education pathway and years of professional experience were also recorded. | Diagnosis | Predicted that the presence of an anchoring diagnosis would influence diagnostic accuracy. Also predicted that participant education pathway and professional experience would influence diagnostic accuracy. | Anchoring effect | N |
| Wilson & Gasek, 1975; USA^142^ | Journal Article | Speech Pathology | Experienced and inexperienced speech clinicians (n = 60) | Participants received the same written pre-information report about a child’s articulation, except that for one half of both groups the report ended with the statement that the child’s articulation problem was mild-to-moderate and for the other half that it was moderate-to-severe. Participants completed a standard articulation inventory based on a video-tape presentation and then rated the child’s articulation on a nine-point scale. | Assessment | Predicted that pre-information would alter participants’ ratings on the standard articulation scale and the nine-point scale, such that the ratings more closely aligned with the pre-information. Also, hypothesised that inexperienced clinicians would be more susceptible to the pre-information bias. | Examiner bias | Y |
| Wisch & Mahalik, 1999; USA^143^ | Journal Article | Psychology | Male therapists (n = 196) | Male therapists were asked to view a series of clinical vignettes that were manipulated for the presenting client variables of sexual orientation and emotional expression. Participants were assessed for sex role conflict and also asked to provide ratings for liking for the client, empathy for the client,  willingness to see the client, client psychological adjustment, client level of functioning, and client prognosis. | Assessment; Prognosis | Predicted that, where clients were depicted as homosexual and violating traditional male gender roles, there would be a negative relationship between participants’ gender role conflict score and ratings for liking for the client, empathy for the client,  willingness to see the client, client psychological adjustment, client level of functioning, and client prognosis. | Sex role stereotype bias; Sexual orientation bias | Y |
| Wolfson et al., 2000; USA^144^ | Journal article | Psychology; Physical Therapy; Occupational Therapy; Speech Pathology | Rehabilitation professionals, including: psychologists; nurses; physical therapists; occupational therapists; speech therapists | Participants undertook a single experimental questionnaire consisting of two sections. Section 1 was designed to assess bias in the use of a functional outcomes instrument (the Functional Independence Measure, FIM). Participants evaluated four hypothetical patient vignettes and provided a FIM score for each item. The four vignettes represented one of two between-sample experimental conditions. All scenarios contained objective information invariant across conditions about patient function. This information was sufficient for identification of an appropriate FIM category. One of two sets of non-essential, but potentially biasing, information was included in each question for 50% of the participants. Participants were randomly assigned to a condition (eg, positive vs negative bias) for each question, and each question was presented in a random order. In Section 2, participants assessed 60 functional descriptions and corresponding FIM ratings, indicating their subjective belief (confidence) that their answers were correct. | Assessment | Predicted that FIM ratings would differ between the vignettes that contained biasing information (age; mental health; prior clinician ratings) and those that did not. | Unspecified biases; Overconfidence | Y |
| Wolfson, 1999; USA^145^ | PhD Dissertation | As above |  |  |  |  |  |  |
| Wolkenstein et al, 2011; Germany^146^ | Journal Article | Psychology | Psychotherapists (n = 204) | Participants were presented with one of six versions of a case vignette describing someone with bipolar and were asked to make a diagnosis. Symptoms (reduced sleep, distractibility) and provision of a causal explanation varied systematically within the vignettes, but each still fulfilled enough diagnostic criteria to be diagnosed as bipolar. The study also assessed the influence of (mis-)diagnosis on therapy recommendations. | Diagnosis; Treatment | Predicted that the addition of ‘reduced sleep’ and ‘distractibility’ symptomology, as well as a lack of causal explanation would influence the likelihood that the participant would diagnose bipolar disorder. Predicted that diagnosis would influence therapy recommendations. | ‘Use of heuristics’ | Y |
| Wood, 2004; USA^147^ | PhD Dissertation | Psychology | Clinical and counselling psychology graduate students (n = 210) | Participants were randomly assigned to one of three diagnostic feedback conditions (principle-based, content-based, control). Participants first read through two training vignettes and were asked to provide diagnostic ratings. Feedback was provided. Participants then provided diagnostic likelihood rating for an additional three vignettes and were asked to provide a confidence rating for their diagnosis. Test vignettes were designed such that symptoms for a conspicuous diagnosis and a less conspicuous overshadowing diagnosis were presented, with both diagnoses presented as an option for which participants were asked to make likelihood ratings for each vignette. | Diagnosis | Predicted that participants in pre-training condition would show overshadowing bias, with differences detected in the average diagnostic likelihood ratings of the conspicuous versus less conspicuous problems. It was also predicted that participants in the training conditions would be more likely to diagnose the overshadowing problem when compared to the control condition for the post-training vignettes. | Diagnostic overshadowing bias | Y |
| Wright & Hutton; 1977; USA^148^ | Journal Article | Psychology | Graduate counsellors (n = 16) | Participants interviewed a female and male actor whom they were led to believe were real clients. To half the clients, the actors posed as upper-middle class and the other half, as lower class. Participants were then asked three questions relating to: the perceived benefit of longer-term treatment; amount of similarity between the participant and client; how much the participant liked the client. Interviews were coded to assess the level of facilitative of the counsellor towards the client. | Prognosis | Hypothesised that the portrayed socioeconomic background of the client would predict differences across each of the measures. | Socioeconomic status bias | N |
| Wu, 2013; USA^149^ | Doctor of Psychology Thesis | Social Work | Child Protection workers and Masters of Social Work students (n = 83) | Participants were randomised to a high risk or low risk anchor condition, and were asked to read one of two vignettes, one describing a family as moderate risk and the other as very high risk. Participants were then further randomised to a control or debiasing condition. Participants undertook risk assessments and were asked to make a recommendation for reunification, both before and after the control/debiasing activity. | Child placement | It was predicted that the risk recommendations would reflect the high and low risk anchor conditions, with participants who read the high risk vignette rating risk as higher and making recommendations for reunification less often. It was also predicted that participants who undertook the debiasing intervention would be less vulnerable to this bias. | Anchoring | N |

*Note:* Three studies were included where the full text was not available because there was sufficient information provided in the abstract to summarise key components of the study relevant to this scoping review.^50, 125, 138^

*Note:* The authors would like to acknowledge that some terms used in older studies, in some cases, would now be considered out of date or potentially offensive by today’s standards. However, for consistency, we have maintained the language that was reported within any given study.
