# Supplementary material for: Decision making biases in the allied health professions: A systematic scoping review
Source: PLoS One. 2020 Oct 20;15(10):e0240716. doi: 10.1371/journal.pone.0240716 (PMC7575084; doi:10.1371/journal.pone.0240716)
Supplement: S5 File — (DOC) [file pone.0240716.s005.doc]

**Supporting Information**

**S5 References (Included Studies)**

1. Abramowitz SI, Roback HB, Schwartz JM, Yasuna A, Abramowitz CV, Gomes B. Sex bias in psychotherapy: a failure to confirm. The American journal of psychiatry. 1976 Jun;133(6):706-9.
2. Abreu JM. Conscious and nonconscious African American stereotypes: impact on first impression and diagnostic ratings by therapists. Journal of consulting and clinical psychology. 1999 Jun;67(3):387.
3. Aronoff, DN. Errors in clinical judgement: The effect of temporal order of client information [dissertation]. [Montreal (Canada)]: McGill University; 1997.
4. Babad EY, Mann M, Mar-Hayim M. Bias in scoring the WISC subtests. Journal of Consulting and Clinical Psychology. 1975 Apr;43(2):268.
5. Barkin SL. *Judgments of professional psychologists about patients with the acquired immunodeficiency disease (AIDS) virus and terminal cancer* [dissertation]. [New York (USA)]: Hofstra University; 1991.
6. Batson CD, Marz B. Dispesitional Bias in Trained Therapists' Diagnoses: Does It Exist? 1. Journal of Applied Social Psychology. 1979 Oct;9(5):476-89.
7. Beasley DS, Manning JI. Experimenter bias and speech pathologists' evaluation of children's language skills. Journal of communication disorders. 1973 Jan 1;6(2):93-101.
8. Becker D, Lamb S. Sex bias in the diagnosis of borderline personality disorder and posttraumatic stress disorder. Professional Psychology: Research and Practice. 1994 Feb;25(1):55.
9. Benefee LM, Abramowitz SI, Weitz LJ, Armstrong SH. Effects of patient racial attribution on black clinicians' inferences. American Journal of Community Psychology. 1976 Sep 1;4(3):263-73.
10. Benjamin SK. Racial Clinical Judgment Bias in Antisocial Personality Disorder Diagnosis [dissertation]. [Connecticut (USA)]: University of Hartford; 2017.
11. Bernstein BL, Lecomte C. Therapist expectancies: Client gender, and therapist gender, profession, and level of training. Journal of Clinical Psychology. 1982 Oct;38(4):744-54.
12. Bieri J, Orcutt BA, Leaman R. Anchoring effects in sequential clinical judgments. The Journal of Abnormal and Social Psychology. 1963 Dec;67(6):616.
13. Billingsley D. Sex bias in psychotherapy: An examination of the effects of client sex, pathology, and therapist sex on treatment planning. Journal of Consulting and Clinical Psychology. 1977 Apr;45(2):250.
14. Bloch PM, Weitz LJ, Abramowitz SI. Racial attribution effects on clinical judgment: A failure to replicate among White clinicians. American Journal of Community Psychology. 1980 Aug 1;8(4):485-93.
15. Brailey K, Vasterling JJ, Franks JJ. Memory of psychodiagnostic information: Biases and effects of expertise. The American journal of psychology. 2001;114(1):55.
16. Bresler JA. *Sex and age bias in clinicians’ and laypersons’ evaluations of mental health psychopathology*[dissertation]. [New York (USA)]: New School for Social Research; 1984.
17. Bruchmüller K, Margraf J, Schneider S. Is ADHD diagnosed in accord with diagnostic criteria? Overdiagnosis and influence of client gender on diagnosis. Journal of consulting and clinical psychology. 2012;80(1):128.
18. Castrianno Galante LM. Subtle racism in child welfare decision-making [dissertation]. [Lincoln (USA)]: University of Nebraska; 1999.
19. Commisso, JT. Errors and biases in clinical judgement [dissertation]. [Pennsylvania (USA]: Temple University; 1994.
20. Curtis HA. Gender bias in the diagnosis of conduct disorder: The effect of client gender on diagnosis, ratings of prognosis, and severity of symptoms [dissertation]. [Newton (USA)]: Boston College; 2002.
21. Crosby JP, Sprock J. Effect of patient sex, clinician sex, and sex role on the diagnosis of antisocial personality disorder: Models of underpathologizing and overpathologizing biases. Journal of Clinical Psychology. 2004 Jun;60(6):583-604.
22. Cwik JC, Papen F, Lemke JE, Margraf J. An investigation of diagnostic accuracy and confidence associated with diagnostic checklists as well as gender biases in relation to mental disorders. Frontiers in psychology. 2016 Nov 22;7:1813.
23. Dailey DM. Are social workers sexists? a replication. Social Work. 1980 Jan 1;25(1):46-50.
24. Dailey DM. Androgyny, sex-role stereotypes, and clinical judgment. InSocial Work Research and Abstracts 1983 Mar 1 (Vol. 19, No. 1, pp. 20-24). Oxford University Press.
25. Delphin ME. Gender and ethnic bias in the diagnosis of antisocial and borderline personality disorders [dissertation]. [West Lafayette (USA)]: Perdue University; 2001.
26. Dewhurst ME, Veach PM, Lampman C, Petraitis J, Kao J, LeRoy B. Probability biases in genetic problem solving: A comparison of undergraduates, genetic counseling graduate students, and genetic counselors. Journal of genetic counseling. 2007;16(2):157-70.
27. Dickert J. Examination of bias in mental health evaluation of deaf patients. Social Work. 1988 May 1;33(3):273-4.
28. Di Nardo PA. Social class and diagnostic suggestion as variables in clinical judgment. Journal of Consulting and Clinical Psychology. 1975 Jun;43(3):363.
29. Diver AJ. Clinical judgement and the use of debiasing techniques: The relationship between accuracy, confidence and the ability to act [dissertation]. [Manchester (England)]: University of Manchester; 1998.
30. Doyle J. Audiologists, the audiogram and the perception of hearing-impaired children’s speech. Australian Journal of Audiology. 1987;9(1):1-6.
31. Eadie T, Sroka A, Wright DR, Merati A. Does knowledge of medical diagnosis bias auditory-perceptual judgments of dysphonia?. Journal of Voice. 2011 Jul 1;25(4):420-9.
32. Egeland B. Examiner expectancy: Effects on the scoring of the wisc. Psychology in the Schools. 1969 Jul;6(3):313-5.
33. Enosh G, Bayer-Topilsky T. Reasoning and bias: Heuristics in safety assessment and placement decisions for children at risk. The British Journal of Social Work. 2014;45(6):1771-87.
34. Fantasia-Davis, RC. The influence of base-rate information in clinical probability judgement [dissertation]. [Bethlehem (USA)]: Lehigh University; 1997.
35. Fernandez RM. Race bias in the diagnosis of schizophrenia [dissertation]. [Terre Haute (USA)]: The College of Graduate and Professional Studies; 2012.
36. Fernbach BE. *Gender differences in diagnosis and treatment recommendations of antisocial personality and somatization disorders* [dissertation]. [Charlottesville (USA)]: University of Viriginia; 1974.
37. Fernbach BE, Winstead BA, Derlega VJ. Sex differences in diagnosis and treatment recommendations for antisocial personality and somatization disorders. Journal of Social and Clinical Psychology. 1989 Sep;8(3):238-55.
38. Fienblatt, JA, Gold, AR. Sex roles and the psychiatric referral process. Sex Roles. 1976;2(2):109-122.
39. Fischer J, Miller H. The effect of client race and social class on clinical judgments. Clinical Social Work Journal. 1973;1(2):100-9.
40. Fischer J, Dulaney DD, Fazio RT, Hudak MT, Zivotofsky E. Are social workers sexists?. Social Work. 1976 Nov 1;21(6):428-33.
41. Ford MR, Widiger TA. Sex bias in the diagnosis of histrionic and antisocial personality disorders. Journal of Consulting and Clinical Psychology. 1989 Apr;57(2):301.
42. Frame RE, Clarizio HF, Porter AC, Vinsonhaler JR. Interclinician agreement and bias in school psychologists' diagnostic and treatment recommendations for a learning disabled child. Psychology in the Schools. 1982 Jul;19(3):319-27.
43. Franklin K, Grossman FM. Bias effects of socioeconomic status and sex in decision making in speech-language pathology. Journal of Speech and Hearing Disorders. 1990 Feb;55(1):82-9.
44. Friedlander ML, Stockman SJ. Anchoring and publicity effects in clinical judgment. Journal of clinical psychology. 1983 Jul;39(4):637-44.
45. Gale TM, Hawley CJ, Butler J, Morton A, Singhal A. Perception of suicide risk in mental health professionals. PloS one. 2016 Feb 24;11(2):e0149791.
46. Gammon RA. Racial and socioeconomic bias in social workers' decisions regarding family reunification [dissertation]. [Alameda (USA)]: California School of Professional Psychology; 2000.
47. Garb HN. The representativeness and past-behavior heuristics in clinical judgment. Professional Psychology: Research and Practice. 1996 Jun;27(3):272.
48. Garfield JC, Weiss SL, Pollack EA. Effects of the child's social class on school counselor's decision making. Journal of Counseling Psychology. 1973 Mar;20(2):166.
49. Garner WA, Strohmer DC, Langford CA, Boas GJ. Diagnostic and treatment overshadowing bias across disabilities: Are rehabilitation professionals immune?. Journal of Applied Rehabilitation Counseling. 1994 Jun 1;25(2):33-7.
50. Goldsmith L, Schloss PJ. Diagnostic overshadowing among learning-disabled and hearing-impaired learners with an apparent secondary diagnosis of behavior disorders. International Journal of Partial Hospitalization. 1984 Sep;*2*(3):209-217.
51. Gomes B, Abramowitz SI. Sex-related patient and therapist effects on clinical judgment. Sex Roles. 1976 Mar 1;2(1):1-3.
52. Gordon TD. Clinical judgment bias in response to client sexual orientation and therapist heterosexuality identity development [dissertation]. [Muncie, Indiana (USA)]: Ball State University; 2010.
53. Gross P. *Clinician Bias Based Upon Client Vocation* [dissertation]. [Los Angeles (USA)]: Alliant International University; 2015.
54. Grupp MG, Glass JL. An examination of possible sexist attitudes among speech-language pathologists toward their clients. Language, Speech, and Hearing Services in Schools. 1980 Jul;11(3):180-7.
55. Haase W. *Rorschach diagnosis socio-economic class, and examiner bias* [dissertation]. [New York (USA)]: New York University; 1956.
56. Hamilton S, Rothbart M, Dawes RM. Sex bias, diagnosis, and DSM-III. Sex Roles. 1986 Sep 1;15(5-6):269-74.
57. Hansen BJ. *Pre-referral observation accuracy and bias of school psychologists* [dissertation]. [Stillwater (USA)]: Oklahoma State University; 2015.
58. Hardy DM, Johnson ME. Influence of therapist gender and client gender, socioeconomic status and alcoholic status on clinical judgments. Journal of Alcohol and Drug Education. 1992; *37*(2): 94-102.
59. Haverkamp BE. Confirmatory bias in hypothesis testing for client-identified and counselor self-generated hypotheses. Journal of Counseling Psychology. 1993 Jul;40(3):303.
60. Hieger B. The influence of diagnostic clarity and prejudicial attitudes on clinical judgment biases [dissertation]. [Pennsylvania (USA)]: Pennsylvania State University; 2007.
61. Hersh JB. Effects of referral information on testers. Journal of Consulting and Clinical Psychology. 1971 Aug;37(1):116.
62. Howell M. Intake decision making in child protective services: Exploring the influence of decision-factors, race and substance abuse [dissertation]. [Richmond (USA)]: Virginia Commonwealth University; 2009.
63. Howells Wrobel NH. Effect of patient age and gender on clinical decisions. Professional Psychology: Research and Practice. 1993 May;24(2):206.
64. Huebner ES, Cummings JA. The impact of sociocultural background and assessment data upon school psychologists' decisions. Journal of School Psychology. 1985 Jun 1;23(2):157-66.
65. Huebner ES. The generalizability of the confirmation bias among school psychologists. School Psychology International. 1990 Nov;11(4):281-6.
66. Jacob CJ. Clinical decision-making in domestic violence scenarios: The influence of experience, information obtained, personal bias, and emotional contagion [dissertation]. [Pennsylvania (USA)]: Pennsylvania State University; 2010.
67. James JW, Haley WE. Age and health bias in practicing clinical psychologists. Psychology and aging. 1995 Dec;10(4):610.
68. Jenkins-Hall K, Sacco WP. Effect of client race and depression on evaluations by white therapists. Journal of Social and Clinical Psychology. 1991 Sep;10(3):322-33.
69. Jenkins MM. *Cognitive de-biasing and the assessment of pediatric bipolar disorder* [dissertation]. [Chapel Hill (USA)]: University of North Carolina; 2012.
70. Jenkins MM, Youngstrom EA. A randomized controlled trial of cognitive debiasing improves assessment and treatment selection for pediatric bipolar disorder. Journal of consulting and clinical psychology. 2016 Apr;84(4):323.
71. Joachim PK. *Diagnostic sex bias in a clinical analogue judgement task* [dissertation]. [New York (USA)]: New York University; 1981.
72. Johannesen, B. Confirmation bias in the evaluation of children’s projective drawings [dissertation]. [Newton (USA)]: Massachusetts School of Professional Psychology; 2014.
73. Jones EE. Psychotherapists' impressions of treatment outcome as a function of race. Journal of Clinical Psychology. 1982 Oct;38(4):722-31.
74. Kolker JI. An investigation of diagnostic sex bias for narcissistic personality disorder, in comparison to histrionic and antisocial personality disorders [dissertation]. [Greensboro (USA)]: The University of North Carolina; 1994.
75. Koscherak S, Masling J. Noblesse oblige effect: The interpretation of Rorschach responses as a function of ascribed social class. Journal of Consulting and Clinical Psychology. 1972 Dec;39(3):415.
76. Langer EJ, Abelson RP. A patient by any other name...: Clinician group difference in labeling bias. Journal of Consulting and Clinical Psychology. 1974 Feb;42(1):4.
77. Lee DY, Richer D, Uhlemann MR. Effects of client preinterview information on counselors' clinical impressions and interview behavior. Counselling Psychology Quarterly. 1992 Apr 1;5(2):115-22.
78. Lee DY, Barak A, Uhlemann MR, Patsula P. Effects of preinterview suggestion on counselor memory, clinical impression, and confidence in judgments. Journal of clinical psychology. 1995 Sep;51(5):666-75.
79. Levy MR, Kahn MW. Interpreter bias on the Rorschach test as a function of patients' socioeconomic status. Journal of Projective Techniques and Personality Assessment. 1970 Apr 1;34(2):106-12.
80. Lowery CR, Higgins RL. Analogue investigation of the relationship between client's sex and treatment recommendations. Journal of consulting and clinical psychology. 1979 Aug;47(4):792.
81. Luepnitz RR, Randolph DL, Gutsch KU. Race and socioeconomic status as confounding variables in the accurate diagnosis of alcoholism. Journal of Clinical Psychology. 1982 Jul;38(3):665-9.
82. Mack GI. *Effects of race on the clinical assessment of antisocial personality disorder and psychopathy* [dissertation]. [New York (USA)]: St John’s University; 1998.
83. Markowitz TB. *The effect of varying order of information on a child custody evaluation* [dissertation]. [Ohio (USA)]: Bowling Green State University; 1989.
84. Martin JM. *Confirmation bias in the therapy session: The effects of expertise, external validity, instruction set, confidence and diagnostic accuracy* [dissertation]. [Memphis (USA)]: University of Memphis; 2000.
85. Matthews J. *The effect of a client’s race/ethnic status and level of acculturation, and the influence of practitioner characteristics, on social workers’ clinical judgements* [dissertation]. [New York (USA)]: Columbia University; 1987.
86. McAshan M. *The impact of client weight and ethnicity on counselors' evaluation of eating disorder symptoms: A vignette study* [dissertation]. [Denton (USA)]: Texas Woman’s University; 2018.
87. McCormick ME. *Can the influence of base rate information be distinguished from the influence of gender stereotypes in the psychiatric diagnostic decision making process?.*[dissertation]. [Rhode Island (USA)]: University of Rhode Island; 2001.
88. Meitus IJ, Ringel RL, House AS, Hotchkiss JC. Clinician bias in evaluating speech proficiency. British Journal of Disorders of Communication. 1973 Jan 1;8(2):146-51.
89. Merluzzi BH, Merluzzi TV. Influence of client race on counselors' assessment of case materials. Journal of Counseling Psychology. 1978 Sep;25(5):399.
90. Messier SH. *Ageism and healthism in diagnosis and prognosis by practising psychologists: An analogue study* [dissertation]. [New York (USA)]: Columbia University; 1997.
91. Michaud LI. *A look at possible bias in the formulation of a psychodiagnosis* [dissertation]. [Kansas (USA)]: University of Kansas; 1994.
92. Mohr JJ, Weiner JL, Chopp RM, Wong SJ. Effects of client bisexuality on clinical judgment: When is bias most likely to occur?.Journal of Counseling Psychology. 2009 Jan;56(1):164.
93. Mosier BC. *Effects of gender bias and gender inversion stereotypes on assessment of personality traits and diagnosis of personality disorders* [dissertation]. [Ruston (USA)]: Louisiana Tech University; 2014.
94. Mumma GH. Effects of three types of potentially biasing information on symptom severity judgments for major depressive episode. Journal of clinical psychology. 2002 Oct;58(10):1327-45.
95. Nalven FB, Hofmann LJ, Bierbryer B. The effects of subjects' age, sex, race, and socioeconomic status on psychologists' estimates of" true IQ" from WISC scores. Journal of clinical psychology. 1969 Jul;25(3):271.
96. New DK. *Observer bias influences in the marschak interaction method rating system* [dissertation]. [Fresno (USA)]: Alliant International University; 2005.
97. O’Reilly CS. *Special education placement decisions: A behavioural decision theory perspective* [dissertation]. [Tucson (USA)]: The University of Arizona; 1986.
98. Oyster-Nelson CK, Cohen LH. The extent of sex bias in clinical treatment recommendations. Professional Psychology. 1981 Aug;12(4):508.
99. Podol J, Salvia J. Effects of visibility of a prepalatal cleft on the evaluation of speech. The Cleft palate journal. 1976 Oct;13:361-6.
100. Parmley MC. *The effects of the confirmation bias on diagnostic decision making* [dissertation]. [Philadelphia (USA)]: Drexal University; 2006.
101. Paster R. *An Exploratory Study of The Influence of Client Race and Ethnicity on Diagnostic Decision-making Processes* [dissertation]. [Boston (USA)]: Massachusetts School of Professional Psychology; 2012.
102. Patterson DR. *Social-cognitive biases in clinical judgement* [dissertation]. [Tallassee (USA)]: Florida State University; 1982.
103. Perlick D, Atkins A. Variations in the reported age of a patient: A source of bias in the diagnosis of depression and dementia. Journal of Consulting and Clinical Psychology. 1984 Oct;52(5):812.
104. Peyser JB. *Examiner bias and intelligence test scoring: a study of the effect of speech-handicapped responses on the scoring of the WISC-R comprehension subtest* [dissertation]. [Berrien Springs (USA)]: Andrews University; 1984.
105. Pfeiffer AM, Whelan JP, Martin JM. Decision-making bias in psychotherapy: Effects of hypothesis source and accountability. Journal of Counseling Psychology. 2000 Oct;47(4):429.
106. Pickholtz HJ. The effects of a child’s racial-ethic label and achievement differences on school psychologists’ decisions [State College (USA)]: Pennsylvania State University; 1977.
107. Prout HT, Frederickson AK. Sex bias in clinical judgment among school psychologists. Psychology in the Schools. 1991 Jul;28(3):226-9.
108. Ramig LA. Effects of examiner expectancy on speech ratings of individuals with cleft lip and/or palate. The Cleft palate journal. 1982 Oct;19(4):270-4.
109. Ray DC, McKinney KA, Ford CV. Differences in psychologists' ratings of older and younger clients. The Gerontologist. 1987 Feb 1;27(1):82-6.
110. Reinsel JM. *An experimental analogue study of the judgment of professional social work practitioners, as influenced by client socio-economic status, worker theoretical orientations, and worker change orientations* [dissertation]. [Columbus (USA)]: Ohio State University; 1976.
111. Roades LA. *Gender and race bias in the diagnosis of major depressive episode and alcohol dependence* [dissertation]. [St Louis (USA)]: University of Missouri; 1994.
112. Robertson J, Fitzgerald LF. The (mis) treatment of men: Effects of client gender role and life-style on diagnosis and attribution of pathology. Journal of Counseling Psychology. 1990 Jan;37(1):3.
113. Rotem-Lehrer N, Singer N, Reshit O, Springer S. Measuring Up to Expectation: Cognitive Bias in Wrist Range-of-Motion Measurement. journal of orthopaedic & sports physical therapy. 2016 Dec;46(12):1037-41.
114. Routh D, King K. Social class bias in clinical judgment. Journal of Consulting and Clinical Psychology. 1972 Apr 1;38(2):202-7.
115. Sattler JM, Hillix WA, Neher LA. Halo effect in examiner scoring of intelligence test responses. Journal of Consulting and Clinical Psychology. 1970 Apr;34(2):172.
116. Saxon JP, Spitznagel RJ. Age bias: An intervening variable in vocational rehabilitation feasibility decision-making?. Journal of Applied Rehabilitation Counseling. 1992;23(1):22-26.
117. Seem SR, Johnson E. Gender bias among counseling trainees: A study of case conceptualization. Counselor Education and Supervision. 1998 Jun;37(4):257-68.
118. Shenkel RJ, Snyder CR, Batson CD, Clark GM. Effects of prior diagnostic information on clinicians' causal attributions of a client's problems. Journal of consulting and clinical psychology. 1979 Apr;47(2):404.
119. Sieracki J. In Whose Best Interest? Using an Experimental Vignette to Assess Factors Influencing Placement Decisions in Child Welfare [dissertation]. [Chicago (USA)]: Loyolo University Chicago; 2010.
120. Smith SM. Evaluating racial bias in inpatient risk assessments [dissertation]. [New York (USA)]: City University of New York; 2013.
121. Snyder C. “A patient by any other name” revisited: Maladjustment or attributional locus of problem?. Journal of Consulting and Clinical Psychology. 1977 Feb 1;45(1):101-3.
122. Spaanjaars NL, Groenier M, van de Ven MO, Witteman CL. Experience and Diagnostic Anchors in Referral Letters. European Journal of Psychological Assessment. 2015;31(4):280-6.
123. Spengler PM, Blustein DL, Strohmer DC. Diagnostic and treatment overshadowing of vocational problems by personal problems. Journal of Counseling Psychology. 1990 Oct;37(4):372.
124. Spengler PM. Does vocational overshadowing even exist? A test of the robustness of the vocational overshadowing bias. Journal of counseling psychology. 2000 Jul;47(3):342.
125. Staal JA. Clinical experience and anchoring effects on therapists' judgement of client mental health [dissertation]. [New Jersey (USA)]: Rutgers The State University of New Jersey; 1993.
126. Steyens G. Bias in the attribution of hyperkinetic behavior as a function of ethnic identification and socioeconomic status. Psychology in the Schools. 1981 Jan;18(1):99-106.
127. Strickland TL, Jenkins JO, Myers HF, Adams HE. Diagnostic judgments as a function of client and therapist race. Journal of Psychopathology and Behavioral Assessment. 1988 Jun 1;10(2):141-51.
128. Swaggerty-Valdes N. *The impact of race and social class on clinician bias* [dissertation]. [Davie (USA)]: Nova Southeastern University; 2009.
129. Tasby CT. Cultural differences and perceptions of autism among school psychologists [dissertation]. [College Station (USA)]: Texas A&M University; 2008.
130. Teitler N. Examiner bias: influence of patient history on perceptual ratings of videostroboscopy. Journal of Voice. 1995 Mar 1;9(1):95-105.
131. Teri L. Effects of sex and sex-role style on clinical judgment. Sex Roles. 1982 Jun 1;8(6):639-49.
132. Trachtman JP. Socio-economic class bias in Rorschach diagnosis: Contributing psychosocial attributes of the clinician [dissertation]. [New York (USA)]: New York University;1968.
133. Umbenhauer SL. *Patient race and social class: Attitudes and decisions among three groups of mental health professionals* [dissertation]. [Cincinnati (USA)]: University of Cincinnati;1975.
134. Vail S. The effects of socio‐economic class, race, and level of experience on social workers’ judgments of clients. Smith College Studies in Social Work. 1970 Jun 1;40(3):236-46.
135. Ventre, J. *The effects of order of presentation of psychoeducational test results and experience level on diagnostic impressions* [dissertation]. [New York (USA)]: Hofstra University; 1987.
136. Waddington L, Morley S. Availability bias in clinical formulation: The first idea that comes to mind. British journal of medical psychology. 2000 Mar;73(1):117-27.
137. Walker BS, Spengler PM. Clinical judgment of major depression in AIDS patients: The effects of clinician complexity and stereotyping. Professional Psychology: Research and Practice. 1995 Jun;26(3):269.
138. Walling CC. *Heuristics in clinical judgment and the labeling theory of mental illness* [dissertation]. [Gainsville (USA)]: University of Florida; 1987.
139. Warner R. The diagnosis of antisocial and hysterical personality disorders. An example of sex bias. The Journal of nervous and mental disease. 1978 Dec;166(12):839.
140. Warner R. Racial and sexual bias in psychiatric diagnosis: psychiatrists and other mental health professionals compared by race, sex, and discipline. The Journal of nervous and mental disease. 1979 May;167(5):303.
141. Wendt DJ, Tyson G. Diagnostic Accuracy in Australian Psychologists: Impact of Experience and Endorsement on the Anchoring Effect. Australian Psychologist. 2018 Jun;53(3):236-42.
142. Wilson WR, Gasek G. The influence of pre-information on the rating of articulation. Journal of communication disorders. 1975 Mar 1;8(1):15-22.
143. Wisch AF, Mahalik JR. Male therapists' clinical bias: Influence of client gender roles and therapist gender role conflict. Journal of Counseling Psychology. 1999 Jan;46(1):51.
144. Wolfson AM. Multidisciplinary physical rehabilitation and the Functional Independence Measure (FIM): Decisional heuristics, biases, and subjective probability analysis [Hattiesburg (USA)]: The University of Southern Mississippi; 1999.
145. Wolfson AM, Doctor JN, Burns SP. Clinician judgments of functional outcomes: how bias and perceived accuracy affect rating. Archives of physical medicine and rehabilitation. 2000 Dec 1;81(12):1567-74.
146. Wolkenstein L, Bruchmüller K, Schmid P, Meyer TD. Misdiagnosing bipolar disorder—do clinicians show heuristic biases?. Journal of affective disorders. 2011 May 1;130(3):405-12.
147. Wood DS. An intervention for diagnostic overshadowing [Tempe (USA)]: Arizona State University; 2004.
148. Wright JA, Hutton BO. Influence of client socioeconomic status on selected behaviors, attitudes, and decisions of counselors. Journal of Counseling Psychology. 1977 Nov;24(6):527.
149. Wu D. Improving child protective services reunification decisions [San Diego (USA)]: Alliant International University; 2013.
