# Supplementary material for: Decision making biases in the allied health professions: A systematic scoping review
Source: PLoS One. 2020 Oct 20;15(10):e0240716. doi: 10.1371/journal.pone.0240716 (PMC7575084; doi:10.1371/journal.pone.0240716)
Supplement: Attachment — Submitted filename: Response to reviewers.docx [file pone.0240716.s006.docx]

**Response to Reviewers (in purple text)**

1. Please ensure that your manuscript meets PLOS ONE's style requirements, including those for file naming. The PLOS ONE style templates can be found at

<https://journals.plos.org/plosone/s/file?id=wjVg/PLOSOne_formatting_sample_main_body.pdf> and

<https://journals.plos.org/plosone/s/file?id=ba62/PLOSOne_formatting_sample_title_authors_affiliations.pdf>

**We have double checked the manuscript against the PLOS ONE style requirements.**

2. Thank you for stating the following in the Competing Interests section:

No authors have competing interests.

We note that one or more of the authors are employed by a commercial company: Redenlab.

1. Please provide an amended Funding Statement declaring this commercial affiliation, as well as a statement regarding the Role of Funders in your study. If the funding organization did not play a role in the study design, data collection and analysis, decision to publish, or preparation of the manuscript and only provided financial support in the form of authors' salaries and/or research materials, please review your statements relating to the author contributions, and ensure you have specifically and accurately indicated the role(s) that these authors had in your study. You can update author roles in the Author Contributions section of the online submission form.

Please also include the following statement within your amended Funding Statement.

“The funder provided support in the form of salaries for authors [insert relevant initials], but did not have any additional role in the study design, data collection and analysis, decision to publish, or preparation of the manuscript. The specific roles of these authors are articulated in the ‘author contributions’ section.”

If your commercial affiliation did play a role in your study, please state and explain this role within your updated Funding Statement.

**The Funding Statement has now been updated.**

2. Please also provide an updated Competing Interests Statement declaring this commercial affiliation along with any other relevant declarations relating to employment, consultancy, patents, products in development, or marketed products, etc.

Within your Competing Interests Statement, please confirm that this commercial affiliation does not alter your adherence to all PLOS ONE policies on sharing data and materials by including the following statement: "This does not alter our adherence to  PLOS ONE policies on sharing data and materials.” (as detailed online in our guide for authors http://journals.plos.org/plosone/s/competing-interests) . If this adherence statement is not accurate and  there are restrictions on sharing of data and/or materials, please state these. Please note that we cannot proceed with consideration of your article until this information has been declared.

Please include both an updated Funding Statement and Competing Interests Statement in your cover letter. We will change the online submission form on your behalf.

**The Competing Interests Statement has now been updated.**


**Reviewer #1**

Reviewer #1: Thank you for the opportunity to review this paper which (as someone with a technical/research interest in the area) was informative in outlining the scope of the literature regarding AHPs and cognitive (and other) biases.

The review was very well written, it was readable, and the scoping review process undertaken diligently - I have no comments to make on the presentation, structure or science behind the paper.

I do however have one major issue with the paper in its current form and some minor points that I think – if addressed – could improve the manuscript.

Taking the major issue first; you have done a good job of describing the who, what and where the biases are in AHPs but you have provided no sense of “so what”? In other words, these biases exist but I (the reader) am none the wiser as to whether they actually matter or not. For example, if I routinely and systematically deny possibly effective therapy [which themselves are subject to considerable uncertainties in effectiveness) to 1:50 of my aboriginal patients is this of the same order of magnitude (clinically, not morally, compared to my colleague who also systematically spends more time and is more conscientious with all their white patients. These are perhaps reductio ad absurdum examples, but hopefully you see my point. There are biases and BIASES… some sense/summary and synthesis of what the effects of these biases are on clinical processes and outcomes, decision success criteria would significantly enhance the sense of “well, that paper mattered” in the reader.
I realise that the studies will be very heterogeneous so I am not suggesting some kind of meta-analysis in the statistical sense, just a guided tour and interpretation of what the effects of these biases on decision quality, outcomes, variations in practice could be.

**Further to the reviewer’s comments, two additional paragraphs have been included in the discussion to more clearly highlight the potentially serious effects that biases may have on clinical decision making, and the implication for patients/clients (line 473). The conclusions have also been revised to highlight the likely negative impacts of such biases (line 523).**

Line 573: “Biases have the potential to seriously impact the quality, consistency and accuracy of decision making in allied health practice.”

Line 473: “Of the total 149 included studies, 77% reported at least one outcome that represented the presence of a bias. It is an assumption made across most of the studies that the presence of bias has a negative impact on the decision making process and consequently the decision outcome for a client/patient. It raises serious concerns that a different clinical decision can be made as a result of a single piece of seemingly irrelevant information (e.g., the ethnic background of the client/patient) which has been included within otherwise identical case scenarios, or as a result of important case information being presented in a different context (e.g., in a different order). That said, most studies did not address whether the decision made was more or less accurate, or whether the decision making process included more errors when a bias was detected. One study^38, 39^ showed that four separate biases were associated with both diagnostic accuracy and decision making error status in the diagnosis of paediatric bipolar disorder. This study also showed that inaccurate, and somewhat accurate, diagnostic decisions were significantly associated with different treatment and clinical recommendations. It will be important for future studies to be able to show that biases result in suboptimal clinical decision making and decision outcomes, leading to inappropriate treatment and/or service provision which results in worse outcomes for clients/patients.

Overall, the body of evidence presented here does suggest that the presence of bias produces inconsistencies in decision making and decision outcomes, and this is a considerable concern in its own right. It indicates that a patient/client may be assessed, diagnosed, and/or receive a different treatment or service as a result of the presence or absence of information that should not have played a role in the decision making process. It is therefore not an unreasonable assumption that cognitive, affective or other biases are likely to have a negative overall effect on the quality of clinical decision making.”

Comparatively minor issues I would like to see addressed include:
1) p4 110-112: I concur with your approach to pulling together a range of biases using systematically altering a decision outcome. However, biases generally come with negative connotations: they systematically alter a decision outcome from some prescriptive/normative idea of “true”/"good"/"quality" (either correspondence - with some empirical criterion or coherence - against some theoretical/logical/probabilistic criterion. if a "bias" systematically alters a decision towards "better" then this isn’t really a problem is it? The negative impact of biases is implicit in your text, but should be made explicit.

**This is a really important point and highly pertinent to the study of biases in healthcare contexts. Many of the studies in the review, particularly the psychology studies, use diagnostic criteria and show that biases, stereotyping for instance, alter the patient/client diagnosis. Whether or not the diagnosis is more or less accurate is unclear. To better determine whether biases have a negative impact on accuracy, diagnostic errors also need to be assessed. Some studies have assessed this, but the majority have not. This point has been included in additional paragraph in the Discussion section (line 473 – see above).**

2) p6 148 – 150: would like to see an assessment of the possible effects of biases on decision process and outcomes in AHPs as an aim (see above)

**A scoping review is largely considered to be a specific type of systematic review, but providing an assessment of effects is beyond the intent of a scoping review. The primary aim of a scoping review is “*to identify knowledge gaps, scope a body of literature, clarify concepts or to investigate research conduct…scoping reviews may also be helpful precursors to systematic reviews and can be used to confirm the relevance of inclusion criteria and potential questions.”*** – Munn Z, Peters MD, Stern C, Tufanaru C, McArthur A, Aromataris E. Systematic review or scoping review? Guidance for authors when choosing between a systematic or scoping review approach. BMC medical research methodology. 2018 Dec 1;18(1):143.

**The present scoping review thus provides an overview of the evidence in this topic area, but as a scoping review, does not intend to provide an assessment of the effects of biases in relation to the specific outcomes reported by the studies. The review does highlight areas where there is sufficient existing evidence to enable a systematic review to be undertaken in future. For instance, the effects of stereotyping biases in psychological diagnosis.**

3) p6 169-172: although AHPs as part of a team could be problematic as “decision making” rarely a solo exercise. The MDT itself likely to introduce different decision processes/reasoning.... c.f. Kenneth Hammond’s idea of the cognitive continuum and the ways in which “visibility” of judgements (i.e. having to explain your rationale/reasoning to MDT colleagues) impacting on reasoning and cue use (and presumably biases). (Hammond, K.R., Human judgement and social policy : irreducible uncertainty, inevitable error, unavoidable injustice. 1996, New York ; Oxford: Oxford University Press.). Perhaps worth flagging in the discussion?

**To address this comment, we have included an additional sentence in the Discussion section flagging that group decision making may further complicate the ability to test bias in decision making in this group of health professionals (line 463).**

Line 463: “This may be further complicated by the reality that many decisions are made by a group of professionals, rather than by an individual.”

4) p7 179-183: very medical range of uncertainties/decision types – which is OK – BUT misses the possibility that AHPs may make additional decisions that lie outwith this typology: c.f. Thompson and nurses and the addition of communication and existential decision types perhaps something to flag as a possible omission/limitation in the discussion <https://ebn.bmj.com/content/7/3/68>

**We specifically did not limit the types of decisions that could be included in this scoping review. We constructed a list of potential examples in our methods of what we expected to find. However, we did not limit study eligibility to this list of examples. We have now included additional wording to this effect in the Methods section (line 234).**

Line 234**: “**Studies reporting on decisions relating to screening, assessment, diagnosis, prognosis and treatment were all eligible, as were studies reporting other decision types not identified here.”

5) p7 outcome measures: how did you control for selection (bias) in studies being reviewed in which the two different groups seeing two different sets of vignettes were not randomly assigned? the study DESIGNS being reviewed could themselves have introduced biases (not cognitive) but impacting on the interpretation or estimation of effects?

**This comment relates to a procedure that would be undertaken for a systematic review through an assessment of the risk of bias in each of the included studies. This analysis is outside of the scope of a scoping review, but would be very relevant to systematic reviews being undertaken in the future on a specific subset of these studies, to answer a more specific research question.**

6) study designs (205-210): RCTs and pre post designs with a control are not the same (in terms of trustworthiness of the results observed... they are of course not invalid but do need to be intellectually adjusted for in the narrative synthesis: ie. higher quality study designs demonstrate lower effects perhaps? Again, needs flagging in discussion for the reader (or detail in the methods if done)

**Please see response to comment #3 above. This point has also been addressed in the paragraph in the Discussion that highlights the limitations of the review (ln 501).**

Overall it’s a thoroughly competent piece but I think you could widen its appeal and interest even further.

**Reviewer #2**

Reviewer #2: Concrete Suggestions:

Line 70: “A lack of consistency in this process can have serious ramifications for patients, clients, and clinicians.” ◊Is consistency the right word? May not require consistency since clinical decision making should cater to the contexts of each individual patient. Nitpicky (and not an error), but would suggest picking a different word. Lack of consistency is arguably necessary.

**We have now replaced the word ‘consistency’ (line 94).**

Line 72: Are any cognitive reasoning processes necessarily inherent? Unclear to me what the distinction between hard wired or learned reasoning processes is, and why they are meaningful.

**This Introduction provides general background relating to cognitive reasoning processes – some biases are thought to arise from cognitive processes that are hardwired (e.g., anchoring effect), and others are thought to be learned and can depend on the environment (e.g., stereotyping). This is an important distinction because the review captures a broad range of biases that would not usually be grouped together as the same ‘type’.**

Define allied healthcare professionals earlier in the paper.

**We have provided a definition earlier in the Introduction section (line 156).**

Line 156: **“**By definition, allied health professionals are health professionals who are not part of the medical, dental or nursing professions. They are university-qualified professionals with specialised expertise in preventing, diagnosing and treating a range of conditions and illnesses.”

Would add a paragraph in the introduction of the paper rationalizing why it’s important to also consider the effect of biases on decisions/outcomes by allied health professionals explicitly. Why the focus on allied health professionals? How are they distinct (or not) from physicians, who seem to be a robust subject of study? Why are they different (or not) from accountants, bankers, loan managers, teachers, cafeteria workers, waiters, screenwriters who all also presumably have sets of biases that affect their work? (I think it would be helpful for the authors to reason their scope of investigation.)

**Further to the reviewer’s recommendation, we have included a further argument for why this review focussed on allied health professions (line 158). The rationale for focussing on this group of health professionals was primarily because much of the work in healthcare has focussed on medical decision making, where some reviews already exist. We wanted to highlight that decision making is equally as important for allied health practitioners, and have focussed on this group of professions because there has not been a review of what research has been done to date. AHPs make important decisions that impact the health and quality of life of their clients/patients and so investigating their decision making is important.**

Line 158: “They are university-qualified professionals with specialised expertise in preventing, diagnosing and treating a range of conditions and illnesses. They make important decisions that impact the health and quality of life of their clients/patients and so investigating their decision making is important. There are obvious parallels in the types of decisions and decision processes undertaken by allied health professionals and medical professionals, and therefore biases likely play an equally significant role.”

I think it would also help if they provided concrete examples of how biases in the allied health professions cause damage/harm/difference in outcome. The reader can generate some through their own imagination, but I think having explicit examples to ground the paper would make it more immediately powerful/applicable/interesting/compelling to readership.

**We have now included additional concrete examples in the introduction (line 164).**

Line 164**: “**For instance: the amount or type of information in a referral letter may alter the type of assessment undertaken or service provided (anchoring bias); a practitioner may be less inclined to alter a prior assessment or diagnosis, and/or change the services currently provided, if key patient/client information is received at a later stage of service delivery (diagnostic momentum; confirmation bias; order effect); the experience of a recent case may influence how a later case is assessed or diagnosed (availability bias; recency effect), or; the physical appearance of a patient or client may alter the assessment or diagnosis, or services provided (stereotyping bias).”

Overall Impression:

This paper meets scientifically rigor and does an excellent job composing a descripting summary of existing research on biases pertaining to allied health professionals. It does give a general view and broad survey, but it also becomes general to the point that the applicability is diluted. Though the paper is informational and does include an explanation of biases and their history, but it doesn’t target or apply to a specific problem. At the end, it reads very broadly, like “there are many biases and they can do many things across outcomes pertaining to allied health professionals,” which is not a particularly compelling conclusion.

As a report on the state of current bias research on allied health professionals, it does the job well. But it’s not particularly satisfying in terms of application, next steps, or what this means for allied health professionals, researchers, or patients—besides the fact that biases are present and they are sometimes studied, though sometimes studied not that well.

The conclusion itself say that it reviews studies that investigate biases, and that further research is needed—especially those that investigate real-life healthcare decision making (instead of using vignettes in the methodology). So while the manuscript perhaps achieves what it sets out to do, it is not a particularly compelling piece of scholarship. Of course, there is a role for research that lays out the state of the state without identifying clear patterns or advocating for specific actions. So, I think it is a clearly designed paper, and well-executed for the purpose that it seeks to achieve (a review looking at what kind of investigations there are regarding biases that affect allied health professionals) but it’s not all that applicable or compelling.

The authors provide many observations. But, without synthesizing strong conclusions from the available literature/existing body of scholarship, it is uncelar what impact this paper would have on addressing clinical biases on the practice of allied health professionals. I do completely agree that the investigation of biases is extremely important, but this manuscript is more of a report than a furthering piece of scholarship, though I recognize that I have my own biases in expecting that scientific research take some stance or advocate for something concrete.

Overall, I think this paper would benefit from a more concrete synthesis instead of spending a bulk of its content on a descriptive summary. Without a stronger conclusion, the reader is left unsure on the take away, which undermines the clarity and purpose.

**We thank the reviewer for their overall support of our scoping review. We would like to clarify that the primary aim of a scoping review is to provide an overview of the current state of research in a particular area (please also see the response to Comment 2) to Reviewer #1, above). We have, however, strengthened the ‘take home’ message of the manuscript by including two additional paragraphs in the Discussion that aims to provide readers with a clearer message that any biases arising in healthcare decision making are likely to have a negative impact on the accuracy and quality of decision making, and this will ultimately impact service delivery (line 474).**
